# Evaluation of TRAP-sequencing technology with a versatile conditional mouse model

**DOI:** 10.1093/nar/gkt995

**Published:** 2013-10-26

**Authors:** Mike Hupe, Minerva Xueting Li, Karin Gertow Gillner, Ralf H. Adams, Jan M. Stenman

**Affiliations:** ^1^Ludwig Institute for Cancer Research Ltd, Box 240, Stockholm SE-171 77, Sweden, ^2^Department of Cell and Molecular Biology, Karolinska Institutet, Stockholm SE-171 77, Sweden and ^3^Max-Planck-Institute for Molecular Biomedicine, Department of Tissue Morphogenesis, and University of Münster, Faculty of Medicine, D-48149 Muenster, Germany

## Abstract

Gene expression profiling of various cell lineages has provided invaluable insights into the molecular mechanisms regulating cellular development and differentiation. However, *in vivo* molecular profiling of rare and interspersed cell populations, such as endothelial cells, has remained challenging. We have generated a versatile floxed translating ribosome affinity purification (TRAP) mouse model, mCherryTRAP, for Cre-dependent translational profiling of distinct cell lineages from intact tissues. To identify cell type–specific transcripts using TRAP, the data have to be filtered to remove both background transcripts not expressed in the profiled cell type and transcripts expressed in all cell populations of the tissue/organ. Filtering has previously been achieved using transcribed RNA from the tissue/organ. Using the mCherryTRAP model, we demonstrate extensive differential expression of RNAs between the translatome and transcriptome of embryonic brains and kidneys. We evaluate the implications of these data for TRAP studies of abundant and rare cell populations. Finally, we demonstrate the applicability of the technology to study organ-specific endothelial cell differentiation.

## INTRODUCTION

Transcriptome analysis using RNA sequencing (RNA-seq) has proven an invaluable tool to better understand complex biological processes. However, many transcripts are subject to posttranscriptional regulation by, for example, microRNAs. Consequently, techniques for translatome analysis, such as ribosome profiling and translating ribosome affinity purification (TRAP), have been developed allowing complementary information about the translational status of the transcripts present in specific cell types to be determined (1–6). The TRAP technology was developed to profile genetically labeled cell populations by purifying translated RNA from intact tissues, thus bypassing the need for lengthy and possibly disruptive cell purification procedures (2). By generating multiple bacterial artificial chromosome transgenic mouse (bacTRAP) models expressing an enhanced green fluorescent protein (EGFP)-tagged ribosomal protein L10a (Rpl10a) fusion protein that incorporates into polysomes, Heiman and colleagues displayed the strengths of the technology by defining unique translational profiles for multiple neuronal cell populations (1,2). Given the large number of available Cre driver lines, the development of a conditional TRAP mouse model would greatly broaden the applicability of the technology. For maximum versatility, this model should offer spatiotemporal control of the expression of fluorescently tagged Rpl10a, driven by a ubiquitous promoter in a well-defined genetic environment.

A key concern in all transcriptome and translatome profiling studies is background RNA from the tissue/organ (7). Transcripts highly expressed in the tissue/organ will appear to be expressed in the profiled cell population even if the contaminating RNA levels are relatively low. For TRAP studies, the optimal approach to overcome this problem is to compare the translated RNA from the profiled cell type with translated RNA from the whole tissue/organ. A fold change can then be calculated for each transcript and those depleted in the cell type–specific translatome can be filtered out as background. Alternatively, those transcripts that are enriched above a certain threshold can be pursued as putative cell type–specific transcripts. Unfortunately, this approach requires the use of two Cre driver lines for activation of the TRAP construct in either the specific cell population or the whole tissue/organ. An alternative approach used in previous TRAP studies is to compare with transcribed RNA from the tissue/organ (1,2,8). However, this method rests on the assumption of little translational regulation, as the fold change for highly translated genes would otherwise be overestimated. The validity of this approach has yet to be formally evaluated.

Here, we report a versatile new tool, the *Gt(ROSA)26Sor-mCherry-Rpl10a* (mCherryTRAP) mouse allele, for studies of *in vivo* translated RNA. Furthermore, we perform an extensive analysis comparing two filtration methods, using either total transcribed RNA (as in previous TRAP studies) or translated RNA from the tissue/organ to identify transcripts enriched in specific cell types. We show that for rare cell populations (brain and kidney endothelial cells), it is reasonable to use transcribed, rather than translated, RNA. However, for abundant cell populations (such as the Emx1-lineage of the dorsal telencephalon), it is important to use translated RNA for identifying enriched transcripts. This conclusion is further supported by gene expression data from the Eurexpress database. Finally, we demonstrate the applicability of TRAP to studies of organ-specific endothelial cell differentiation.

## MATERIALS AND METHODS

### Animals and genotyping

Animal care and research protocols were in accordance with institutional guidelines, and approved by the Etiska Nämnden on animal use. For staging of embryos, the morning of vaginal plug was designated as E0.5. *Sox2Cre* (9), *Emx1Cre* (10) and Tg(Cdh5-cre/ERT2)1Rha *(Cdh5CreERT2*) (11) mice and embryos were genotyped by polymerase chain reaction (PCR) using the following primers: *Sox2Cre*-forward CTC TAG AGC CTC TGC TAA CC, *Sox2Cre*-reverse CCT GGC GAT CCC TGA ACA TGT CC, or *genericCre*-forward CAC GAC CAA GTG ACA GCA AT, *genericCre-*reverse AGA GAC GGA AAT CCA TCG CT.

To generate the *Gt(ROSA)26Sor-mCherry-Rpl10a* allele, we PCR amplified *mCherry* from mCherry-pRSET-B (provided by Roger Tsien) using the following primers: NheI-mCherry-5 AAA CCC GCT AGC GCC GCC ACC ATG GTG AGC AAG GGC GAG G and XhoI-mCherry-3 AAA CCC CTC GAG ATC TTG TAC AGC TCG TCC ATG C, and *Rpl10a* from CS-EGFP-L10A (provided by Nathaniel Heintz) using XhoI-Rpl10a-5 TCA GAT CTC GAG CTC AAG CTT and NotI-Rpl10a-3 GGG AAA GCG GCC GCC TAA TAC AGA CGC TGG GGC T. *mCherry* was digested with NheI/XhoI, *Rpl10a* with XhoI/NotI, and subcloned into then NheI/NotI sites of pBSApBpACAGftILn (12). Finally, the conditional expression construct was released with PacI and AscI, and subcloned into the PacI and AscI sites of pRosa26PAS (13). The construct was linearized and electroporated into F1 ES cells (14). Colonies were screened by PCR using the following primers: Rosa26-5armFlanking CCT AAA GAA GAG GCT GTG CTT TGG and Rosa26-SA CAT CAA GGA AAC CCT GGA CTA CTG. Positive colonies were expanded and confirmed by PCR. One targeted clone was injected into host (C57BL/6J, Jackson Laboratories) blastocysts by the Karolinska Center for Transgene Technologies, Karolinska Institutet. Mice and embryos were genotyped using the following primers: R26-mCherry-Rpl10a-forward TAC ACC ATC GTG GAA CAG TAC, R26-mCherry-Rpl10a-reverse GTA GTT CTT CAG GCT GAT CTG, R26-wt-forward GCG GAT CAC AAG CAA TAA TA and R26-wt-reverse TTT CTG GGA GTT CTC TGC TG. Tamoxifen (Sigma) was resuspended in corn oil (20 mg/ml) and administered by oral gavage before harvesting embryos.

### Histological analysis

Embryos were harvested at E14.5, fixed for 3 h in 4% paraformaldehyde, rinsed thoroughly in phosphate buffered saline (PBS), submerged in 30% sucrose, embedded in optimal cutting temperature (OCT) medium and finally frozen in a dry ice/ethanol bath. Tissue cryosections (10–12 μm) were collected on slides for histological analysis. Adult brains were removed fresh, immersion fixed in 4% paraformaldehyde in PBS overnight at 4°C and then placed in PBS with 30% sucrose for at least 72 h at 4°C before sectioning on a cryostat. The adult brains were sectioned at 30–40 μm and kept as free-floating in PBS. For immunohistochemistry, slide-mounted sections were incubated in a blocking/permeabilization solution containing 10% donkey serum and 0.25% Triton X-100 for 1 h at room temperature, followed by incubation in primary antibody solution overnight at 4°C. The sections were incubated with appropriate secondary antibodies conjugated to Alexa fluorophores for 2 h at room temperature before mounting in Immu-Mount (Thermo Scientific). The adult brain sections were stained free-floating and subsequently mounted onto slides. Confocal microscopy was performed on a Zeiss LSM510 confocal microscope. We analyzed a minimum of three embryos for each genotype. Primary antibodies used were: rat anti-CD144 (BD Biosciences, 550548, 1:500), rat anti-CD31 antibody (BD Biosciences, 553370, 1:500) and rabbit anti-dsRed antibody (Clontech, 632496, 1:500).

### Immunoblotting

Standard methods for immunoblotting were used: Tissue was homogenized in ice-cold lysis buffer [20 mM HEPES (pH 7.4), 150 mM KCl, 5 mM MgCl_2_, 0.5% Nonidet P-40 (NP-40) and protease inhibitors (Roche)] with a hand glass homogenizer. Homogenates were incubated at 4°C with end-over-end rotation for 10 min. Cellular debris were removed by centrifugation at 16 100*g* for 10 min. Proteins was supplemented with 4-fold concentrated Laemmli buffer and boiled at 95°C for 5 min. Proteins were separated by 10% sodium dodecyl sulphate-polyacrylamide gel electrophoresis, followed by transfer to nitrocellulose membranes using iBlot® Gel Transfer (Invitrogen). Membranes were blocked with 5% nonfat milk/PBS-T (PBS-0.1% Tween-20) for 1 h, washed with PBS-T and incubated in PBS-T containing 2.5% nonfat dry milk and primary antibodies as indicated overnight. After washing in PBS-T, blots were incubated with horseradish peroxidase-conjugated secondary antibody in 1% nonfat milk/PBS-T for 1 h. After washing blots with PBS-T, bands were visualized with enhanced chemiluminescence detection kit (Thermo Scientific). Antibodies were used as follows: anti-DsRed: 632496, Clontech, 1:2000 in 2.5% nonfat milk/PBS-T; anti-Rpl7: ab72550, Abcam, 1:5000 in 2.5% nonfat milk/PBS-T.

### Purification of mRNA from mCherryTRAP mice

Embryos were harvested and scored for mCherry expression using a fluorescence microscope. The brain (forebrain and part of the diencephalon) and kidneys were dissected in ice-cold PBS. Respective tissue from one to two embryos was immediately homogenized in ice-cold polysome extraction buffer [20 mM HEPES (pH 7.4), 150 mM KCl, 5 mM MgCl_2_, 0.5% NP-40, 0.5 mM dithiothreitol, 100 mg/ml cycloheximide (Sigma), protease inhibitors (Roche) and 40 U/ml recombinant RNase inhibitor (Promega)] with a hand glass homogenizer. Homogenates were incubated at 4°C with end-over-end rotation for 10 min. Subsequently, crude extracts were cleared by three centrifugation steps (2600, 8600 and 16 100*g* each for 5 min at 4°C). Anti-RFP magnetic beads (M165-9, MBL), two times washed with polysome extraction buffer, were added to the supernatant, and the mixture was incubated at 4°C with end-over-end rotation for 30 min. Beads were subsequently collected on a magnetic rack and washed four times with high-salt polysome wash buffer [20 mM HEPES (pH 7.4), 350 mM KCl, 5 mM MgCl_2_, 0.5% NP-40, 0.5 mM dithiothreitol, 100 mg/ml cycloheximide, protease inhibitors and 40 U/ml recombinant RNase inhibitor]. RNA was eluted from the beads by incubating beads in RLT buffer (Rneasy® Micro Kit, Qiagen) + 10 μl/ml β-mercaptoethanol for 5 min at room temperature. Eluted RNA was purified using RNeasy® Micro Kit (Qiagen) following the manufacturer’s instructions including in-column DNase digestion.

### Quantitative PCR

Purified RNA (20 ng) was used to produce cDNA with a Maxima® First Strand cDNA Synthesis Kit for RT-qPCR (Thermo Scientific) following the manufacturer’s instructions. Resulting cDNA was used to perform the quantitative PCR with Platinum® SYBR® Green qPCR SuperMix-UDG (Invitrogen), with 500 nM final concentration of each primer. Cycling and quantitation were performed with ViiA^TM^ 7 Real-Time PCR System (Applied Biosystems) using the ViiA^TM^ 7 software v1.2. PCR was carried out for 2 min 50°C, 2 min 95°C, 40 cycles (15 s 95°C, 30 s 60°C), followed by a melt curve. Each replicate was assayed in triplicate. Data are normalized to *Rpl19* and presented as mean ± SEM. The following primers were used: *Cdh5*-fwd CAA TGA CAA CTT CCC CGT CT, *Cdh5*-rev CGT TTG GGG TCT GTC TCA AT, *Chd5*-fwd TCT AGC CGT CGT CGT GAC TT, *Chd5*-rev CAA CAC CGC ACC AAC TCC T, *Pecam1*-fwd AGA GAC GGT CTT GTC GCA GT, *Pecam1*-rev TAC TGG GCT TCG AGA GCA TT, *Rpl19*-fwd GGT GAC CTG GAT GAG AAG GA, *Rpl19*-rev TTC AGC TTG TGG ATG TGC TC, *Slc2a1*-fwd AAC TTC ATT GTG GGC ATG TG, *Slc2a1*-rev GAA GCG ATC TCA TCG AAG GT, *Sox3*-fwd TGG GAC CGT TGC CTT GTA CCG, *Sox3*-rev GTC CCA TTT CCG CTG CTC GGG, *Tubb3*-fwd GCG CCT TTG GAC ACC TAT T and *Tubb3*-rev TTC CGC ACG ACA TCT AGG AC.

### RNA-seq and analysis

The RNA quality was validated using the Agilent RNA 6000 Pico or Nano Kit and an Agilent 2100 bioanalyzer (Agilent Technologies, Inc.). RNA (100 ng–1 μg) was used to generate RNA-seq libraries with the TruSeq™ RNA Sample prep kit v2 kit (Illumina) following the manufacturer’s protocol. The sequencing libraries were validated using the Agilent DNA 1000 kit and Agilent bioanalyzer. All samples had an average size ∼300 bp. Equal amounts of three to four indexed sequencing libraries were pooled and diluted to a final concentration of 2 nM, and sequenced on an Illumina platform. Sequence reads were mapped against the *Mus musculus* genome assembly (Genome Reference Consortium GRCm38, UCSC version mm10) using the RUM v. 2.04 pipeline (15). Gene expression (reads per kilobase per million mapped reads, RPKM) calculations were performed using rpkmforgenes.py (16). The mm10 refGene.txt-file was downloaded from UCSC 20130114. Differential expression analysis was performed with DESeq (17). Gene ontology was performed with DAVID 6.7 (18,19). RSeQC was used for gene body read coverage analysis (20). Linear regression was performed using the scipy.stats.linregress (least-squares regression) function.

## RESULTS AND DISCUSSION

### Generation and functional validation of the mCherryTRAP mouse model

**Figure 1.**
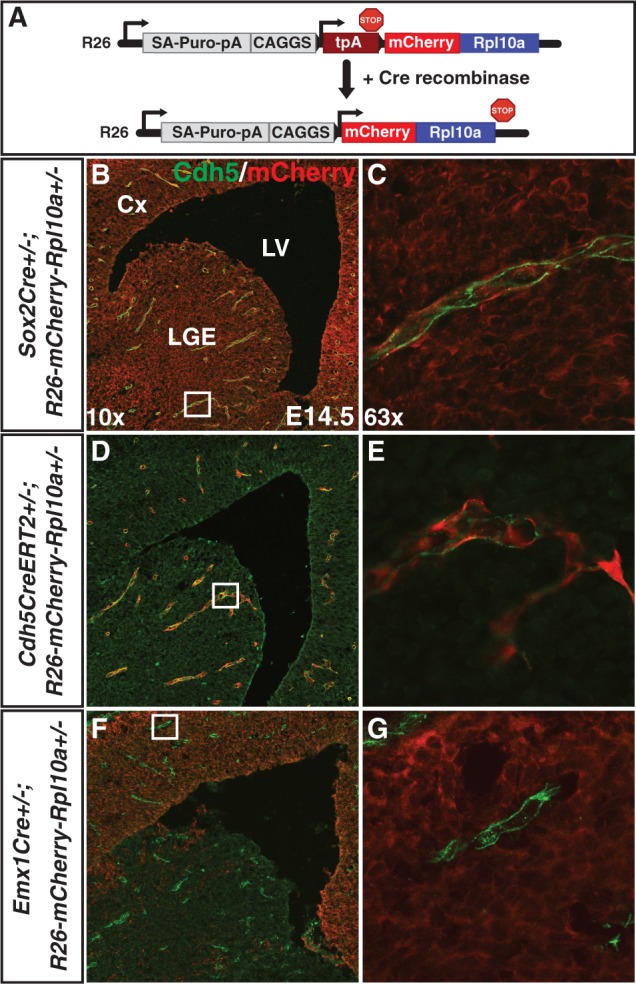
A Cre-dependent mCherryTRAP mouse model. (**A**) Schematic of the *Gt(ROSA)26Sor-mCherry-Rpl10a* allele before and after Cre mediated recombination. In the presence of Cre recombinase, the stop cassette (tpA; three SV40 polyAs) is recombined out and the ubiquitous enhancer/promoter (CAGGS) drives expression of mCherry-tagged Rpl10a. (**B–G**) Three Cre driver mouse lines were crossed with the *R26-mCherry-Rpl10a* line to specifically activate expression of *mCherry-Rpl10a* in (B and C) all cells of the embryo proper (*Sox2Cre*), (D and E) endothelial cells (*Cdh5CreERT2*) or (F and G) neural cells in the dorsal telencephalon (*Emx1Cre*) at E14.5. mCherry fluorescence (B and C) and immunofluorescence using antibodies directed against Cdh5 (green; endothelial cells) and dsRed (red; D–G) demonstrate mCherry-Rpl10a protein expression in (B and C) all cells, (D and E) endothelial cells, or (F and G) neural cells in the E14.5 telencephalon. Note that mCherry-Rpl10a protein is not detected in brain endothelial cells in *Emx1Cre+/−; R26-mCherry-Rpl10a+/−* embryos. The boxes in (B, D and F; 10× magnification) show the regions in (C, E and G; 63× magnification), respectively. Cx, Cortex; LGE, lateral ganglionic eminence; LV, lateral ventricle.

To increase the number of cell lineages amenable to TRAP, we developed the floxed mCherryTRAP mouse model. We first generated a mCherry-Rpl10a fusion protein rather than using the EGFP-Rpl10a fusion protein from previous studies, reasoning that it will offer important advantages: First, it will provide maximum compatibility with existing GFPCre and GFPCreERT2 driver lines. Second, it will allow researchers to profile two separate cell populations from the same material by taking advantage of the battery of available bacTRAP transgenic mice. The conditional expression cassette was introduced into the well-characterized *Gt(ROSA)26Sor* locus (R26 locus; Figure 1A). Robust expression and proper cellular distribution of mCherry-Rpl10a (driven by the ubiquitous CAGGS enhancer/promoter) was observed in embryonic stem cells only after Cre-mediated recombination (Supplementary Figure S1). The established mCherryTRAP mouse line was crossed with various Cre driver lines, *Cdh5CreERT2* (endothelial cells) (11), *Sox2Cre* (highly efficient recombination in all epiblast derivatives) (9) and *Emx1Cre* (dorsal telencephalon) (10) to demonstrate Cre-dependent expression of mCherry-Rpl10a in specific cell populations of embryonic day (E)14.5 (Figure 1B–G) and adult (Supplementary Figure S2) brains.

Endothelial cells constitute a rare cell population that is interspersed between other cell types, making it hard to purify and profile. To test our mCherryTRAP mouse line, *Cdh5CreERT2* was used to activate expression of the mCherry-Rpl10a fusion protein in endothelial cells. Embryos for RNA extraction were selected by visual inspection using a fluorescence microscope, and commercially available precoupled anti-RFP magnetic beads were used to immunoprecipitate polysomes and translated RNA from intact E14.5 forebrains (Supplementary Figure S3). Enrichment of brain endothelial cell markers (*Cdh5, Pecam1, Slc2a1*) and depletion of neural lineage markers (*Chd5, Sox3, Tubb3*) was consistently observed, thus demonstrating functionality of the conditional mCherryTRAP mouse model.

### Evaluation of TRAP-seq for molecular profiling of rare and abundant cell populations

**Figure 2.**
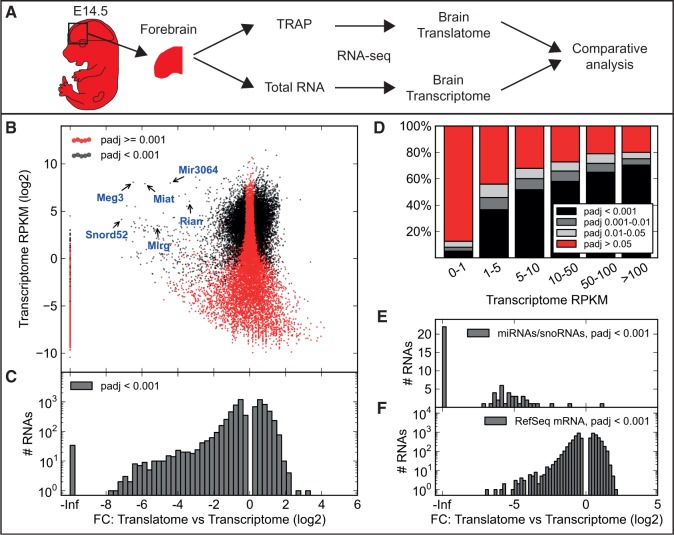
Differential expression of transcripts between the embryonic brain translatome and transcriptome. (**A**) Schematic of the experimental procedure to compare the brain translatome and transcriptome. Red color shows tissues expressing the mCherry-Rpl10a fusion protein. (**B**) Scatter plot of the fold change (FC) between translated (immunoprecipitated) and transcribed RNA from *Sox2Cre+/−; R26-mCherry-Rpl10a+/−* E14.5 forebrains versus the average RPKM value for the transcript in the brain transcriptome reveals RNA-specific differential expression. padj—adjusted *P*-value. Six noncoding transcripts are highlighted demonstrating that they all, as expected, are depleted in the brain translatome. (**C**) Histogram of the fold changes for RNAs determined to be differentially expressed, suggesting that more RNAs are weakly than highly translated. (**D**) Stacked bar chart showing the percentage of RNAs in different expression level intervals (average RPKM in the brain transcriptome) that are not significantly regulated (red) or regulated (light gray, dark gray, black). Note that by statistical analysis 72.6% of the RNAs expressed >5 RPKM are scored as differentially expressed between the translatome and transcriptome. (**E** and **F**) Histograms of the fold change for micro- (mi) and small nucleolar (sno) RNAs (E) and RefSeq mRNAs (F). miRNAs and snoRNAs are not translated and therefore depleted after TRAP. More mRNAs are weakly than highly translated.

In previous TRAP studies, identification of transcripts with cell type–specific expression was achieved by calculating a fold-enrichment between the translated RNA from the specific cell type and total RNA from the tissue/organ (1,2,8). To further examine the validity of the assumption of this approach, i.e. close to a one-to-one correspondence between transcribed and translated transcripts, we set out to compare the translatomes with the transcriptomes of E14.5 brains and kidneys (Figure 2A and Supplementary Figure S4A). The *Sox2Cre* driver line was used to achieve activation of the TRAP-construct in all cells of the embryo proper (9). We then dissected E14.5 forebrains and kidneys, purified both translated and transcribed RNA from each of three independent biological replicates per organ and performed Illumina RNA-seq [Supplementary Table S1; TRAP followed by RNA-seq is referred to as TRAP-seq (3)]. Importantly, the reproducibility between replicates was high (Supplementary Figures S5 and S6), and similar gene body coverage profiles were obtained from the translatome and transcriptome samples (Supplementary Figure S7).

Translated RNAs constitute a subset of the total cell RNA pool. Therefore, highly translated transcripts should be relatively more abundant in the translatome than the transcriptome, and this would thus be reflected in the number of reads and the calculated expression values. Differential expression analysis of our RNA and TRAP-seq data [using DESeq (17)] revealed significant differences between the translatome and transcriptome expression values for a large number of specific RNAs both in brain (Figure 2B–F) and kidney (Supplementary Figure S4B–F). Our data show that ∼70% of the transcripts with an RPKM [0.3–1 transcripts/RPKM (21,22)] over 5 are differentially expressed between the translatome and transcriptome (Figure 2D and Supplementary Figure S4D), suggesting extensive translational regulation. Consistent with previous reports, which have shown that short transcripts have higher ribosome density and correlate with higher translational activity (5,23), we found that shorter transcripts are enriched in the brain and kidney translatome (Supplementary Figure S8). However, transcript length appears to be a weak predictor of translational activity. Furthermore, as expected, most microRNAs and small nucleolar RNAs are missing or significantly reduced in the TRAP samples (Figure 2B and E and Supplementary Figure S4E), further validating the functionality of our mCherryTRAP allele and the methodology. In addition, the long noncoding transcripts, *Meg3* (24), *Rian* (25), *Mirg* (26) and *Miat* (27) are highly enriched in the brain transcriptome (Figure 2B). Interestingly, 85 RefSeq noncoding transcripts appear to be enriched in the translated RNA from the brain (60 in the kidney), suggesting that they are coding mRNAs (Supplementary Figure S9). Most RefSeq mRNAs are moderately enriched or depleted (±4-fold change; Figure 2F and Supplementary Figure S4F), suggesting that filtering TRAP-seq data obtained from distinct cell populations with transcribed RNA obtained from the whole tissue/organ is likely to result in fold change values with up to a 4-fold error for many transcripts. As the theoretical maximum fold change values is lower for transcripts in abundant cell types, we predict more difficulties identifying transcripts specifically enriched in such cell populations.

**Figure 3.**
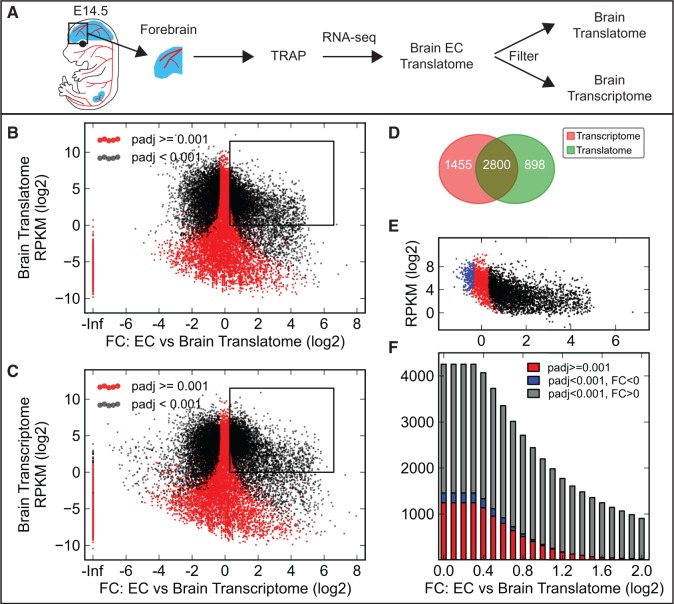
Evaluation of TRAP-seq by molecular profiling of brain endothelial cells. (**A**) Schematic of the experimental procedure to profile brain endothelial cells. Two methods for identifying cell type–specific transcripts are compared, using either translated or transcribed RNA from the forebrain. Red color shows tissues expressing the mCherry-Rpl10a fusion protein. (**B** and **C**) Scatter plots of the fold change (FC) between immunoprecipitated endothelial cell (EC) RNA from E14.5 *Cdh5CreERT2+/−; R26-mCherry-Rpl10a+/−* forebrain and translated (B) or transcribed (C) RNA from the entire E14.5 *Sox2Cre+/−; R26-mCherry-Rpl10a+/−* forebrain versus the average RPKM value for the transcript in the translatome (B) or transcriptome (C). Many transcripts are strongly enriched (positive fold change) in brain endothelial cells. (**D**) Venn diagram showing the number of RNAs identified as enriched in brain endothelial cells compared with the brain translatome (box in B) or transcriptome (box in C) RNA (only RNAs >1 RPKM). (**E**) All RNAs identified as brain endothelial cell enriched in the comparison with brain transcriptome (C) were plotted using the fold change value obtained in the comparison with translatome RNA (B). Several RNAs were predicted by the transcriptome comparison to be enriched in endothelial cells, while in fact not significantly regulated (red, 1242 genes) or even reduced (blue, 213 genes). (**F**) A gradually increasing FC threshold was applied to the set of RNAs predicted to be enriched in brain endothelial cells by the transcriptome comparison, and the number of RNAs that were (gray) or were not (red and blue) enriched also in the translatome comparison were quantified (see also Supplementary Figure S11).

**Figure 4.**
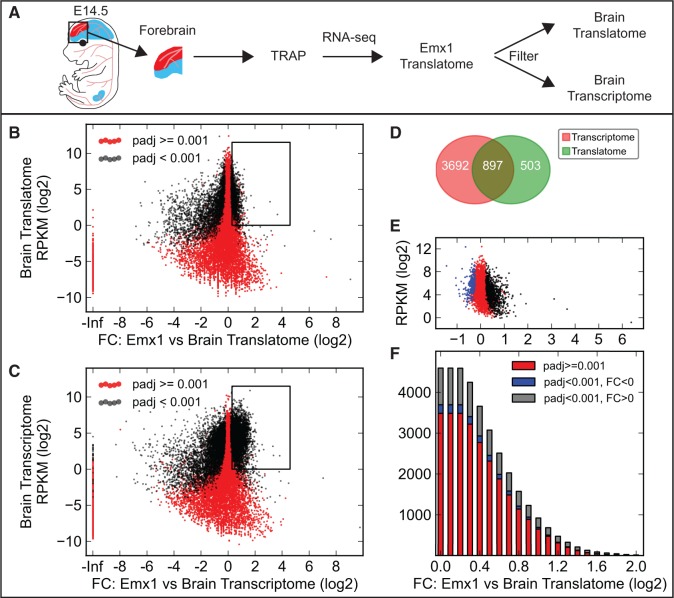
Evaluation of TRAP-seq by molecular profiling of the Emx1-lineage in the dorsal telencephalon. (**A**) Schematic of the experimental procedure to profile the Emx1-lineage in the dorsal telencephalon. Two methods for identifying cell type–specific transcripts are compared, using either translated or transcribed RNA from the forebrain. Red color shows tissues expressing the mCherry-Rpl10a fusion protein. (**B** and **C**) Scatter plots of the fold change (FC) between immunoprecipitated dorsal telencephalon (Emx1) RNA from E14.5 *Emx1Cre+/−;R26-mCherry-Rpl10a+/−* forebrains and translated (B) or transcribed (C) RNA from the entire E14.5 *Sox2Cre+/−;R26-mCherry-Rpl10a+/−* forebrain versus the average RPKM value for the transcript in the translatome (B) or transcriptome (C) RNA. Note that fewer genes are predicted to be enriched in the Emx1-positive dorsal telencephalon in (B) than in (C). (**D**) Venn diagram showing the number of RNAs identified as enriched in Emx1 cells compared with the translatome (box in B) or transcriptome (box in C) RNA (only RNAs >1 RPKM). (**E**) All RNAs identified as Emx1-cell lineage enriched in the comparison with the brain transcriptome (C) were plotted using the fold change value obtained in the comparison with translatome RNA (B). A majority of the genes that were predicted to be enriched by the transcriptome comparison were in fact not significantly regulated (red, 3485 genes) or even reduced (blue, 207 genes) in the translatome comparison. (**F**) A gradually increasing FC threshold was applied to the set of RNAs predicted to be enriched in Emx1-positive cells of the dorsal telencephalon by the transcriptome comparison, and the number of RNAs that were (gray) or were not (red and blue) enriched also in the translatome comparison were quantified (see also Supplementary Figure S11).

To further test this prediction, *mCherry-Rpl10a* was activated in three cell populations: brain and kidney endothelial cells (*Cdh5CreERT2*) and the Emx1-lineage in the dorsal telencephalon (*Emx1Cre*). The brain and kidney endothelial cell populations are rare in relation to the total number of cells in the organs in which they are found. In contrast, the Emx1-positive cell lineage is an abundant cell population in the telencephalon with a maximum theoretical fold change only around two. Forebrains and kidneys were dissected from fluorescent embryos, and the affinity purified RNA was sequenced (Figures 3A and 4A and Supplementary Figure S10A). Again, a high reproducibility between the biological replicates was found (Supplementary Figures S5 and S6). A differential expression analysis comparing the cell type–specific RNA with both the transcribed and translated RNA previously obtained from E14.5 forebrains and kidneys demonstrated that many transcripts are strongly enriched in brain (Figure 3B and C) and kidney (Supplementary Figure S10B and C) endothelial cells. 2800 transcripts expressed >1 RPKM were by statistical analysis identified as enriched in brain endothelial cells by both filtration methods. However, 1455 transcripts were only identified when filtering with the brain transcriptome. The majority of these transcripts were not significantly regulated when compared with the brain translatome, and some were even significantly depleted (Figure 3D and E; kidney endothelial cells; Supplementary Figure S10D and E). In contrast and as predicted, almost no transcripts exhibit a fold change higher than two in the telencephalic Emx1-lineage (Figure 4B and C). Of the 4589 transcripts identified as enriched in the telencephalic Emx1-lineage when filtering with the brain transcriptome, only 897 transcripts were also identified by the translatome filtration method (Figure 4D and E).

The number of transcripts scored as differentially expressed between the translatome and transcriptome of E14.5 brains (and kidneys) decrease when increasing the required minimum fold change value (Figure 2 and Supplementary Figure S4). In fact, we identified few transcripts with fold change values higher than four. This suggests that focusing only on the cell type enriched transcripts with high fold change values will increase the specificity of the transcriptome filtering method. To test this hypothesis, we first identified all brain (and kidney) endothelial cell or Emx1-lineage transcripts with fold change values against the transcriptome above specific thresholds. We then asked what fold changes these transcripts exhibited when compared with the tissue translatome and whether these were statistically significant (Figures 3F and 4F and Supplementary Figures S10F and S11). For brain endothelial transcripts with a fold change higher than four, 903 transcripts were identified by the transcriptome comparison. Merely 23 of these transcripts were not significantly upregulated in the translatome comparison (kidney endothelial cells: 6 of 455). However, for the Emx1-lineage, only 18 genes were identified by the transcriptome comparison, and 8 of these were not significantly upregulated in the translatome comparison. Thus, increasing the fold change has dramatically reduced the sensitivity without a major improvement of the specificity. Interestingly, this means that for rare cell populations, such as brain and kidney endothelial cells, transcript filtration can be performed using the tissue/organ transcriptome as the gene sets identified using both methods are similar. However, for more abundant cell populations, our data suggest that the translatome filtering approach should be used.

**Figure 5.**
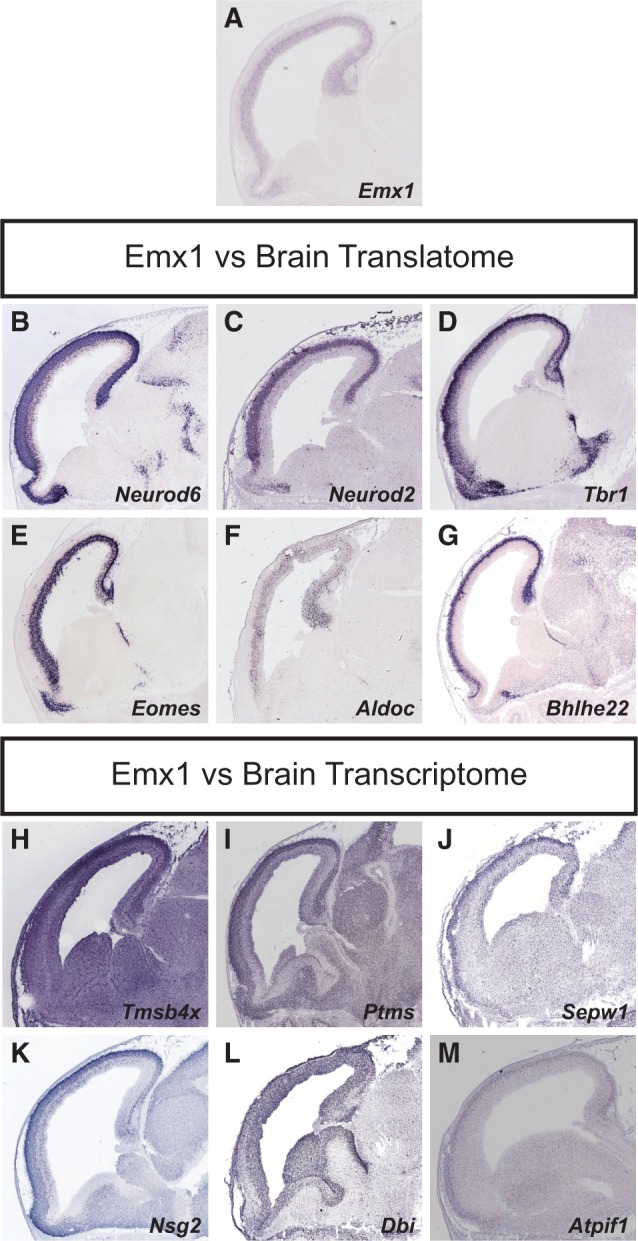
Gene expression pattern of the top six highest expressed transcripts identified as enriched in the Emx1-lineage versus the brain translatome or transcriptome. E14.5 gene expression data from Eurexpress (used with permission) is shown for *Emx1* (**A**) and the top 6 highest expressed genes identified as enriched in the Emx1-lineage versus the brain translatome (**B–G**) or transcriptome (**H–M**) with a fold change >2 (adjusted *P* < 0.001). Note that the *Emx1 in situ* only shows current expression (A), and that the Emx1-lineage also includes cells that historically have expressed *Emx1*. All six transcripts identified as enriched in the Emx1-lineage versus the brain translatome are clearly enriched in the dorsal telencephalon (B–G). In contrast, all six transcripts identified as enriched in the Emx1-lineage versus the brain transcriptome are not enriched in the dorsal telencephalon or are inconclusive (H–M). See also Supplementary Figures S12 and S13.

To provide further experimental validation in support of our claim, we used the digital transcriptome atlas Eurexpress [www.eurexpress.org (28)], which documents the gene expression pattern of thousands of genes at E14.5. We examined the expression pattern of the 25 highest expressed transcripts identified as enriched in the Emx1-lineage using either the transcriptome or the translatome filtering approach (fold change >2). We found that 13 of the transcripts identified as enriched compared with the translatome were clearly enriched in the Emx1-lineage, seven were not enriched or were inconclusive and five were not present in the database (Figure 5 and Supplementary Figure S12 and Supplementary Table S2). However, only one of the transcripts identified after transcriptome filtering was clearly enriched in the dorsal telencephalon, and the rest were either not enriched or inconclusive (Figure 5 and Supplementary Figure S13 and Supplementary Table S3). Thus, we conclude that for the more abundant cell populations, the tissue/organ translatome should be used.

We wanted to determine how the TRAP method compares with other profiling techniques. Therefore, we compared our brain endothelial cell translational profile with published transcriptional profiles obtained by mouse thiouracil (TU) tagging (29) or fluorescence-activated cell sorting (FACS) (30). Thirteen positive control genes were selected for validation (29,30). Importantly, TRAP is performing favorably with FACS (Table 1). TU tagging identifies fewer transcripts, and the fold enrichments were much weaker. However, TU tagging is complementary to other transcriptional and translational profiling methods as it isolates cell type–specific nascent RNA. To further evaluate our TRAP-seq results, a gene ontology analysis of the transcripts identified to be enriched in brain or kidney endothelial cells was performed. This analysis revealed that terms such as ‘blood vessel development’ and ‘angiogenesis’ were strongly enriched (Supplementary Figure S14 and Supplementary Tables S4–S7). In conclusion, TRAP-sequencing is a convenient and highly efficient technology for *in vivo* profiling of rare cell populations, such as endothelial cells, that are hard to purify.

**Table 1.** Molecular profiling of brain endothelial cells using FACS, TU tagging or TRAP

Comparison of profiling techniques				
(Fold enrichment in endothelial cells)				
Genes	TRAP	TU tagging		FACS
	E14.5	E15.5	P6	P60–70
*Cdh5*	24.7	4.7	2.8	28.7
*Cd34*	33.9	5.9	1.8	13.7
*Egfl7*	22.1	1.0	3.1	29.9
*Emcn*	31.2	4.6	3.3	20.4
*Esam*	25.8	1.8	3.5	13.8
*Ets1*	20.1	2.4	4.7	37.0
*Flt1*	18.4	3.5	5.2	40.8
*Kdr*	15.4	1.8	5.6	27.1
*Nos3*	18.9	2.1	3.4	14.4
*Pecam1*	30.0	3.9	3.3	21.9
*Tek*	21.4	3.4	3.6	25.9
*Tie1*	32.3	2.6	1.4	32.0
*Thsd1*	21.3	1.5	4.4	12.9

Thirteen genes previously identified as enriched in brain endothelial cells using FACS or TU tagging. The values indicated are the fold changes and are calculated as average RPKM of brain EC RNA/average RPKM of brain transcriptome (TRAP); average RPM of TU-tagged RNA/average RPM of total RNA (TU tagging); expression value for CNS endothelial cells/expression value for CNS parenchyma for the probe set with the strongest enrichment (FACS). Note that TRAP compares favorably with FACS, while TU tagging identifies fewer transcripts with much weaker fold enrichments.

### TRAP to study organ-specific endothelial cell differentiation

To properly serve the needs of distinct organs, the endothelial cells that line the blood vessels display striking molecular, morphological and functional heterogeneity (31–33). Thus, the fenestrated endothelium of the kidney allows efficient filtration of the blood. In contrast, the brain endothelium forms a tight barrier, the blood–brain barrier, to limit passive diffusion of solutes from the blood, and thus protects the sensitive neural cells. The molecular mechanisms regulating organ-specific endothelial cell development are still poorly understood, partly due to the difficulties in accessing the cells for profiling experiments. Therefore, we wanted to ascertain whether TRAP could reliably be used for comparative translatome studies of distinct endothelial cell populations.

**Figure 6.**
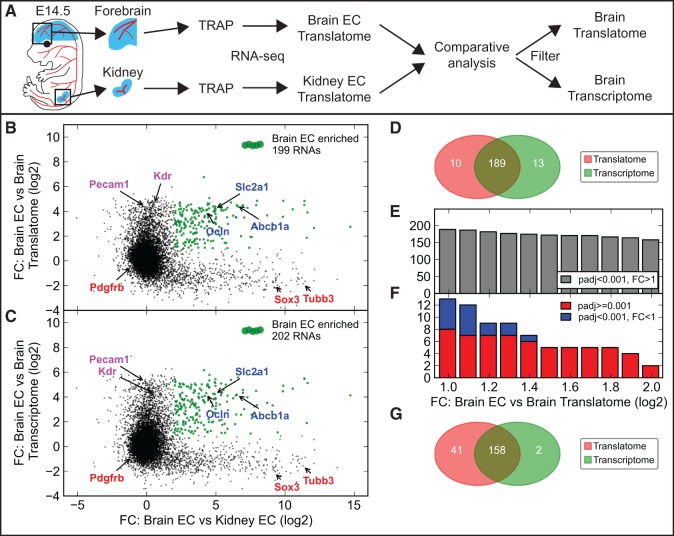
Validation of the applicability of TRAP-seq to study organ-specific brain endothelial cell differentiation. (**A**) Schematic of the experimental procedure to compare the molecular profile of brain and kidney endothelial cells. Red color shows tissues expressing the mCherry-Rpl10a fusion protein. (**B** and **C**) Scatter plots of the fold change (FC) between immunoprecipitated endothelial cell (EC) RNA from E14.5 *Cdh5CreERT2+/−;R26-mCherry-Rpl10a+/−* forebrains and kidneys versus the FC between brain EC RNA and translated (B) or transcribed (**C**) RNA from E14.5 *Sox2Cre+/−;R26-mCherry-Rpl10a+/−* forebrains. RNAs that are at least 2-fold enriched versus the brain translatome (B) or transcriptome (**C**), and 4-fold enriched in brain versus kidney EC are indicated with green dots (*n* = 199 in B, *n* = 202 in C). General EC markers (magenta), pericyte and neural markers (red), and three well-known brain-enriched EC markers (blue) are indicated. (**D**) Venn diagram comparing the RNAs identified as brain EC markers from the analyses in (B and C). 13 transcripts were predicted to be brain EC enriched by the transcriptome, but not the translatome, analysis. (**E** and **F**) A gradually increasing FC threshold (brain EC versus brain transcriptome) was applied to the set of RNAs predicted to be brain EC enriched by the transcriptome comparison, and the number of RNAs that were (gray in E) or were not (red and blue in F) enriched also in the translatome comparison were quantified. (**G**) A Venn diagram showing that with a FC threshold >4, the number of transcripts identified as brain enriched after transcriptome filtering have dropped from 202 to 160, but only two of these were not identified by the translatome comparison.

We compared the translational profiles of brain and kidney endothelial cells, focusing on transcripts with a minimum RPKM of 5 exhibiting at least 4-fold higher expression levels in one of the organs (Figure 6 and Supplementary Figure S15). We identified enriched transcripts using either the established brain/kidney transcriptomes or translatomes (Figure 6A–C and Supplementary Figure S15A–C), starting by requiring a fold change higher than two. This analysis identified 199 (translatome) or 202 (transcriptome) transcripts enriched in brain versus kidney endothelial cells, and 80 (translatome) or 84 (transcriptome) transcripts in kidney versus brain endothelial cells (Figure 6D and Supplementary Figure S15D and Supplementary Tables S8 and S9). Thirteen of the 202 transcripts identified by filtering with transcribed RNA were not identified when filtering with translated RNA. Further increasing the required fold change compared with transcribed RNA to four noticeably increases the specificity (Figure 6E–G and Supplementary Figure S15E–G). This demonstrates the applicability of TRAP-sequencing to studies of organ-specific endothelial cell differentiation using the organ transcriptome to identify cell type–specific transcripts.

## CONCLUSION

Our floxed mCherryTRAP mouse line provides scientists in diverse research areas with a versatile tool for Cre-dependent translational profiling of distinct cell lineages from intact tissues. As whole tissues, or even organs, are used as input for TRAP, it is a convenient method for *in vivo* profiling of rare cell types that are hard to purify by, for example, microdissection or cell sorting. Our study has important implications for the design of TRAP experiments that aim to define molecular profiles for specific cell populations. We show that it is essential to consider the abundance of the cell type in the tissue/organ. For rare cell populations, using the transcriptome, or translatome, of the entire tissue/organ as filters to identify those transcripts enriched in the specific cell type results in similar transcript lists. However, for more abundant cell populations, it appears necessary to use the translatome. Our finding was further supported by gene expression data from the Eurexpress database. Unfortunately, this approach requires the use of two Cre driver lines: one cell lineage specific, and one for achieving recombination in all cells.

The need for different filtration methods can be understood by considering the large number of transcripts that exhibited fold change differences of up to four between the organ transcriptomes and translatomes. While the maximum fold change values are higher than four for transcripts in the more rare endothelial cell populations, they are only around two for the transcripts in the more abundant Emx1 cell lineage in the forebrain. The differences observed between the trancriptomes and translatomes may entirely, or partly, reflect translational regulation due to, for example, uncoupling of transcription and translation in the highly proliferative cells of the embryo. In adults, who mostly consist of postmitotic cells, the correlation between transcribed and translated transcripts may be higher. However, we cannot completely rule out that the differences observed between our transcriptome and translatome data, at least partly, are caused by biases inherent in the method. For example, the activity of the CAGGS enhancer/promoter may differ between cell states or cell types, thus resulting in different levels of mCherry-Rpl10a fusion protein. Alternatively, if the endogenous levels of Rpl10a vary between cell types, different levels of the mCherry-Rpl10a fusion protein may be required to outcompete it and label the same percentage of ribosomes. While it is clear from our analysis that TRAP can be used to identify known noncoding transcripts, it remains to be firmly established what percentage of the other differentially expressed transcripts identified in our work truly are translationally regulated. To fully resolve this question, a comparison of the organs’ transcriptomes and translatomes using alternative techniques, such as polysome fractionation or ribosome profiling, and a careful analysis of the biases of the respective techniques will be necessary. However, even in the unlikely case that the differences between our transcriptome and translatome data sets are mainly caused by biases inherent in the TRAP methodology, our finding that the abundance of the cell type is an important consideration when designing molecular profiling experiments remains valid.

Finally, our TRAP-seq data sets provide interesting opportunities for future studies. For example, are the multiple RefSeq noncoding transcripts identified as enriched in the translatome actually coding mRNAs? How is translation regulated during brain and kidney development? Can different transcription factor codes be defined for the brain and kidney endothelial cells, and can these transcription factors be used to reprogram other cells types into brain and kidney endothelial fates?

## ACCESSION NUMBERS

The reported RNA-seq data have been deposited in NCBI’s Gene Expression Omnibus and are accessible through GEO Series accession number GSE51619 (http://www.ncbi.nlm.nih.gov/geo/query/acc.cgi?acc=GSE51619).

## SUPPLEMENTARY DATA

Supplementary Data are available at NAR Online.

## FUNDING

Wenner-Gren Stiftelserna, postdoctoral fellowship (to M.H.); Wenner-Gren Fellows (to J.M.S.); Cancerfonden [CAN 2010/679]. Funding for open access charge: Ludwig Institute for Cancer Research Ltd.

*Conflict of interest statement*. None declared.

## Supplementary Material

Supplementary Data
