# Supplementary material for: Evaluation of TRAP-sequencing technology with a versatile conditional mouse model
Source: Nucleic Acids Res. 2013 Oct 26;42(2):e14. doi: 10.1093/nar/gkt995 (PMC3902954; doi:10.1093/nar/gkt995)
Supplement: Supplementary Data [file supp_42_2_e14__index.html]

Evaluation of TRAP-sequencing technology with a versatile conditional mouse model — Evaluation of TRAP-sequencing technology with a versatile conditional mouse model — Supplementary Data 

# Evaluation of TRAP-sequencing technology with a versatile conditional mouse model

## Supplementary Data

files

**Files in this Data Supplement:**

- Supplementary Data - pdf file
- Supplementary Data - xls file
