# Supplementary material for: Evaluation of TRAP-sequencing technology with a versatile conditional mouse model
Source: Nucleic Acids Res. 2013 Oct 26;42(2):e14. doi: 10.1093/nar/gkt995 (PMC3902954; doi:10.1093/nar/gkt995)
Supplement: Supplementary Data [file supp_gkt995_nar-01722-met-k-2013-File010.pdf]

Supplementary Figure S1: Distribution of mCherry-Rpl10a protein in embryonic stem (ES) cells.

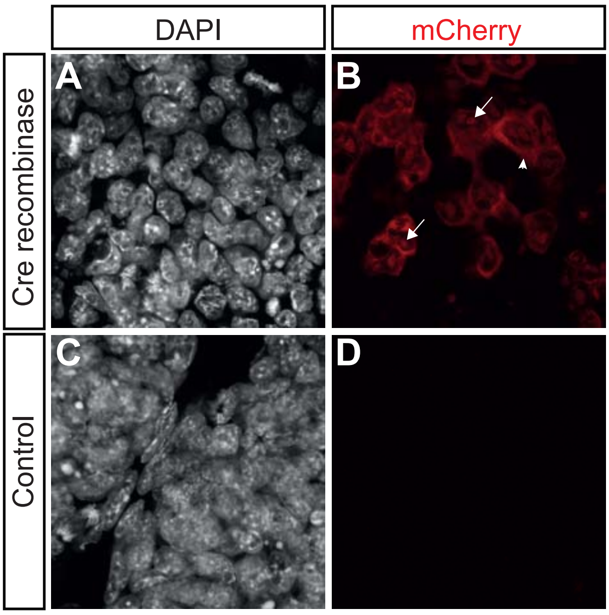

R26-mCherry-Rpl10a knock-in ES cells were (**A**, **B**), or were not (**C**, **D**), transiently transfected with a Cre recombinase expression construct and counterstained with DAPI (gray; **A**, **C**). mCherry fluorescence (red; **B**) confirms expression and localization of the mCherry-Rpl10a fusion protein. Note the nucleolar (arrows) and cytoplasmic (arrowhead) distribution of mCherry-Rpl10a consistent with incorporation into polysomes. (**C**, **D**) No leakage was detected in the absence of Cre protein.

Supplementary Figure S2: Conditional expression of mCherry-Rpl10a in adult forebrain endothelial cells.

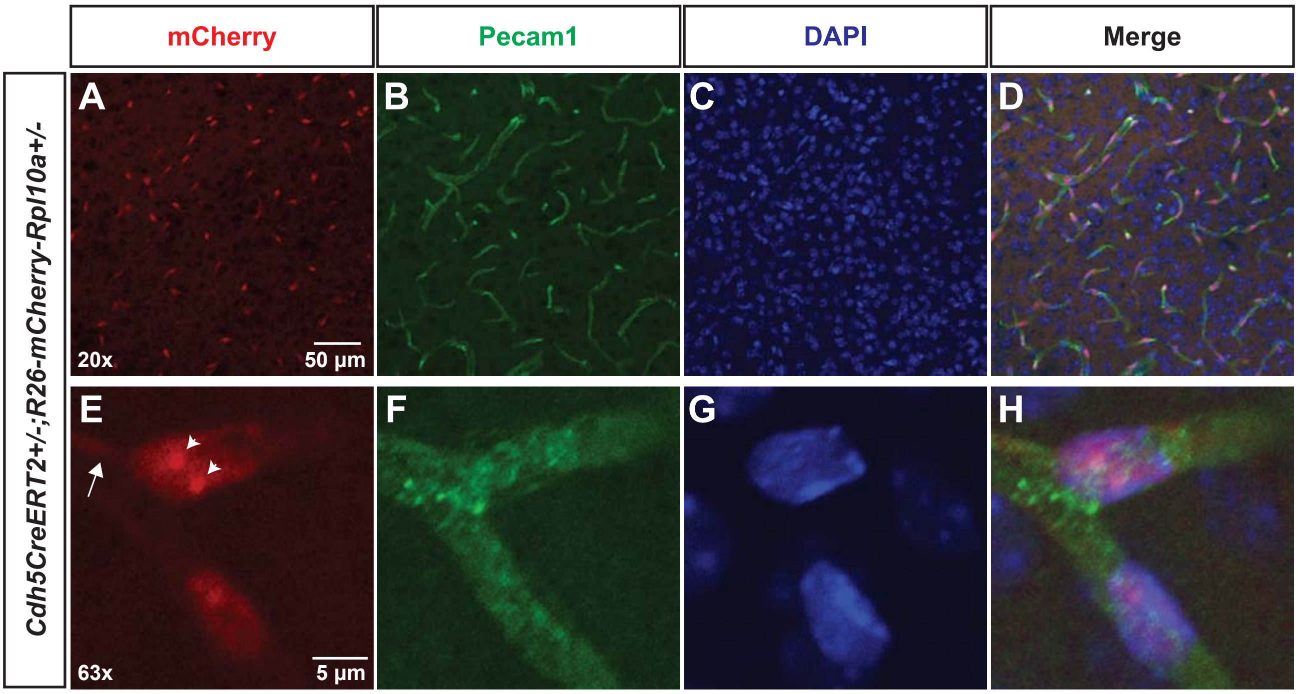

mCherry fluorescence (red; **A**, **D**, **E**, **H**), immunofluorescence using an antibody directed against Pecam1 (green; **B**, **D**, **F**, **H**), and DAPI nuclear counterstaining (blue; **C**, **D**, **G**, **H**) demonstrates presence of the mCherry-Rpl10a fusion protein in endothelial cells of an adult *Cdh5CreERT2+/-; R26-mCherry-Rpl10a+/-* mouse forebrain (**A**, **B**, **C**, **D** - 20x magnification, **E**, **F**, **G**, **H** - 63x magnification). Note the nucleolar (arrowheads) and cytoplasmic (arrow) distribution of mCherry-Rpl10a consistent with incorporation into polysomes also in vivo.

Supplementary Figure 3: Verification of the functionality of the mCherryTRAP mouse line.

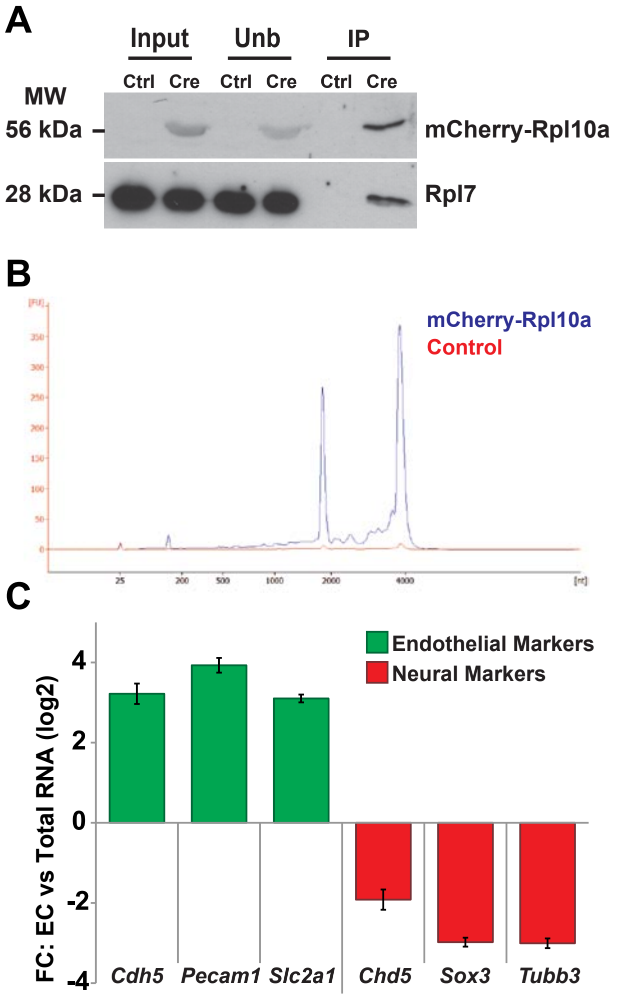

(**A**) Representative immunoprecipitation of mCherry-tagged Rpl10a and co-immunoprecipitation of untagged ribosomal protein L7 (Rpl7) from E14.5 *Cdh5CreERT2+/-;R26-mCherry-Rpl10a+/-* (Cre), but not control (Ctrl), forebrains. mCherry-Rpl10 – Input 2%; Unb, 2% unbound; IP, 20% immunoprecipitated sample; Rpl7 – Input 0.2%; Unb 0.2%; IP 20%. (**B**) Representative purification of RNA from E14.5 *Cdh5CreERT2+/-;R26-mCherry-Rpl10a+/-* (blue), but not control (red), forebrains as detected by Bioanalyzer PicoChips (Agilent Technologies). FU – fluorescence units, nt – nucleotides. (**C**) qRT-PCR analysis of affinity purified brain endothelial RNA (EC) from *Cdh5CreERT2+/-;R26-mCherry-Rpl10a+/-* E14.5 forebrains reveals an enrichment of brain endothelial cell markers (*Cdh5*, *Pecam1*, *Slc2a1*) and a reduction of neural lineage markers (*Chd5*, *Sox3*, *Tubb3*). Values are mean +/- SEM of three biological replicates. FC – fold change.

Supplementary Figure S4: Differential expression of transcripts between the embryonic kidney translatome and transcriptome.

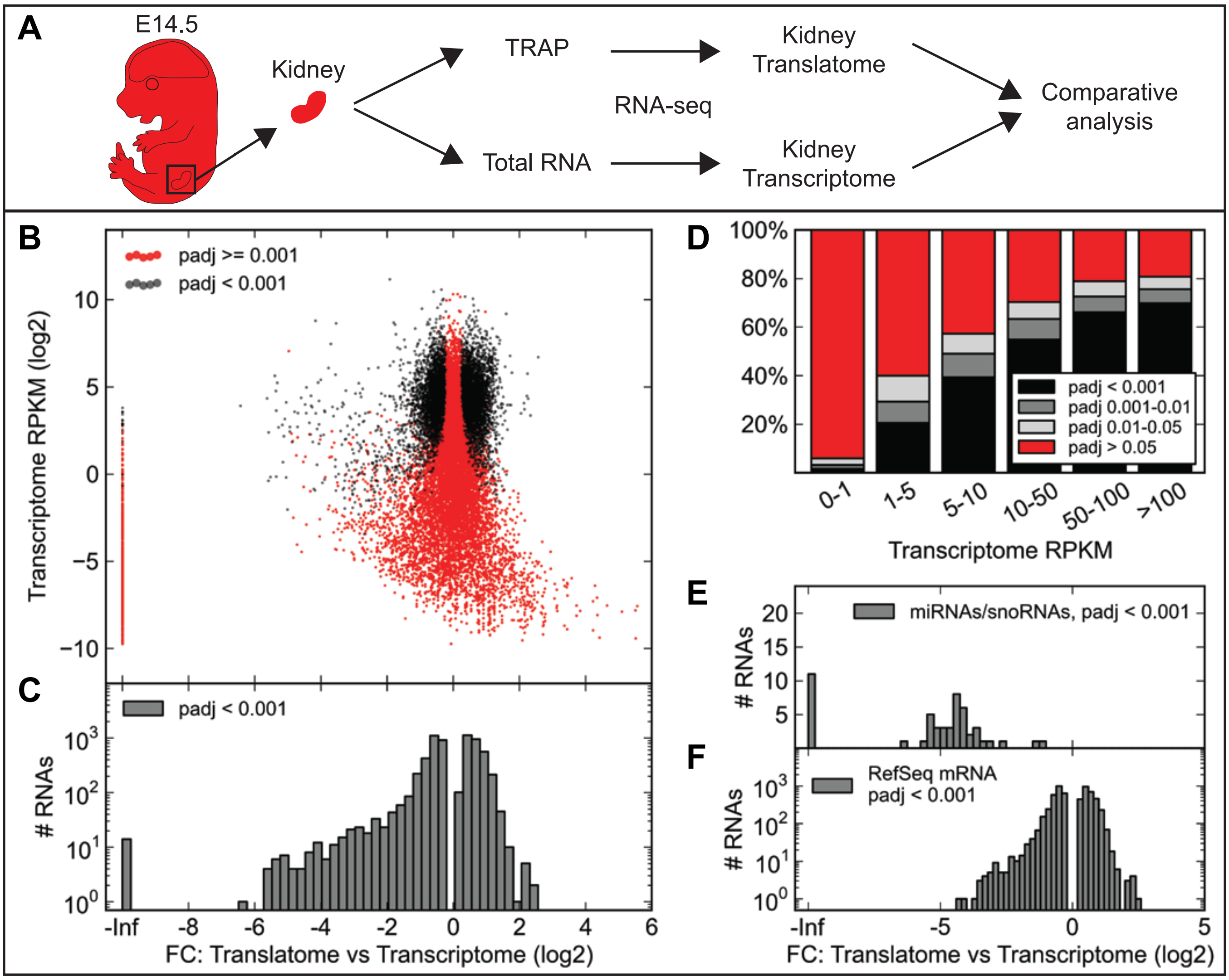

(**A**) Schematic of the experimental procedure to compare the kidney translatome and transcriptome. Red color shows tissues expressing the mCherry-Rpl10a fusion protein. (**B**) Scatter plot of the fold change (FC) between translated (immunoprecipitated) and transcribed RNA from *Sox2Cre+/-;R26-mCherry-Rpl10a+/-* E14.5 kidneys versus the average RPKM value for the transcript in the kidney transcriptome reveals RNA-specific differential expression. padj - adjusted p-value. (**C**) Histogram of the fold changes for RNAs determined to be differentially expressed, suggesting that more RNAs are weakly than highly translated. (**D**) Stacked bar chart showing the percentage of RNAs in different expression level intervals (average RPKM in the kidney transcriptome) that are not significantly regulated (red) or regulated (light grey, dark grey, black). Note that by statistical analysis 69.0% of the RNAs expressed higher than 5 RPKM are scored as differentially expressed between the translatome and transcriptome. (**E**, **F**) Histograms of the fold change for micro- (mi) and small nucleolar (sno) RNAs (**E**), and RefSeq mRNAs (**F**). miRNAs and snoRNAs are not translated and therefore depleted after TRAP. More mRNAs are weakly than highly translated.

Supplementary Figure S5: Log-log plots of biological replicates.

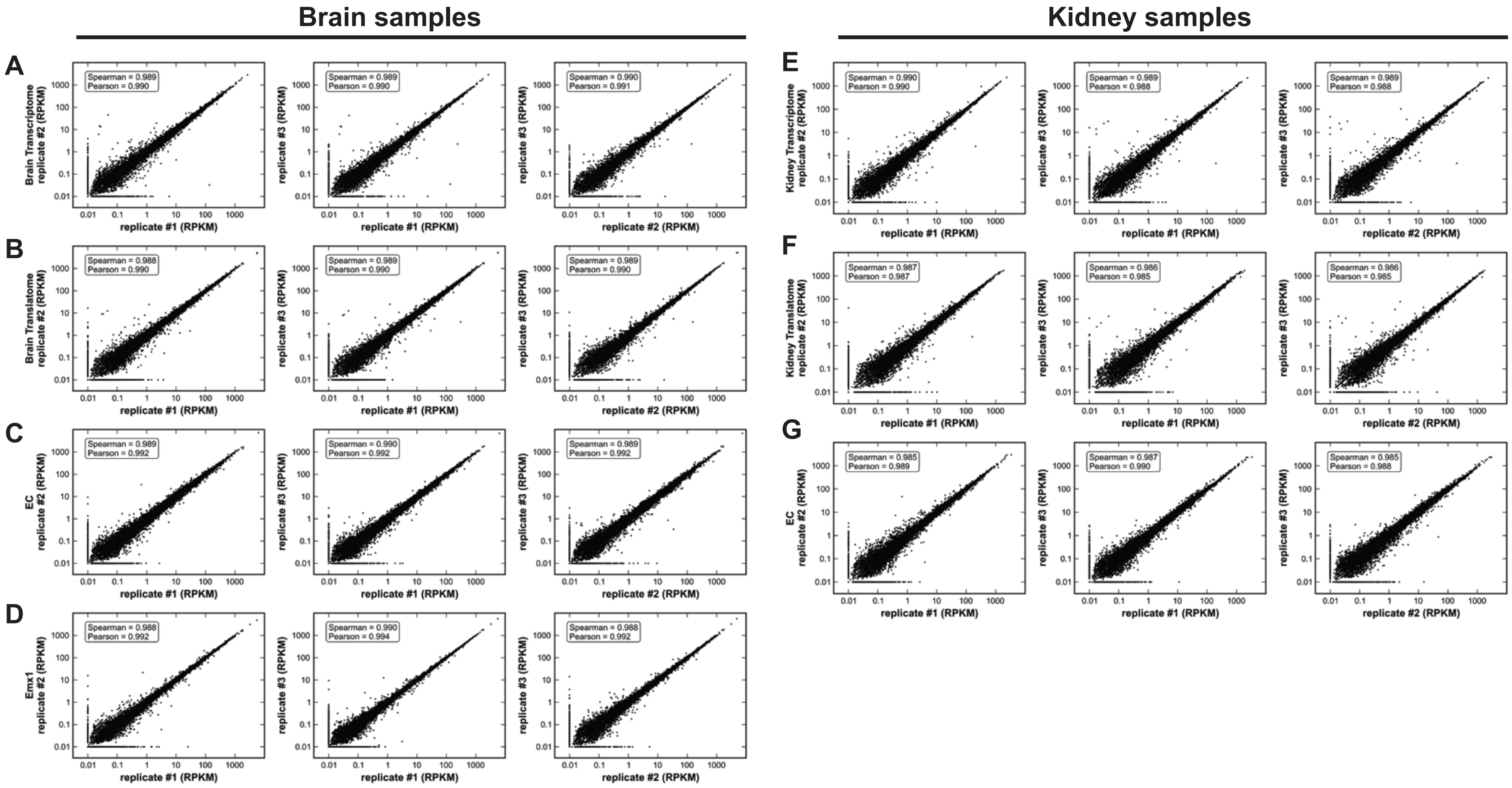

Log-log plots of RPKM-values demonstrate high reproducibility between the three biological replicates for all brain and kidney transcribed RNA (**A**, **E**) and TRAP (**B**, **C**, **D**, **F**, **G**) samples. The Pearson product-moment correlation coefficients and Spearman's rank correlation coefficients are shown.

Supplementary Figure S6: Principal component analysis of biological replicates.

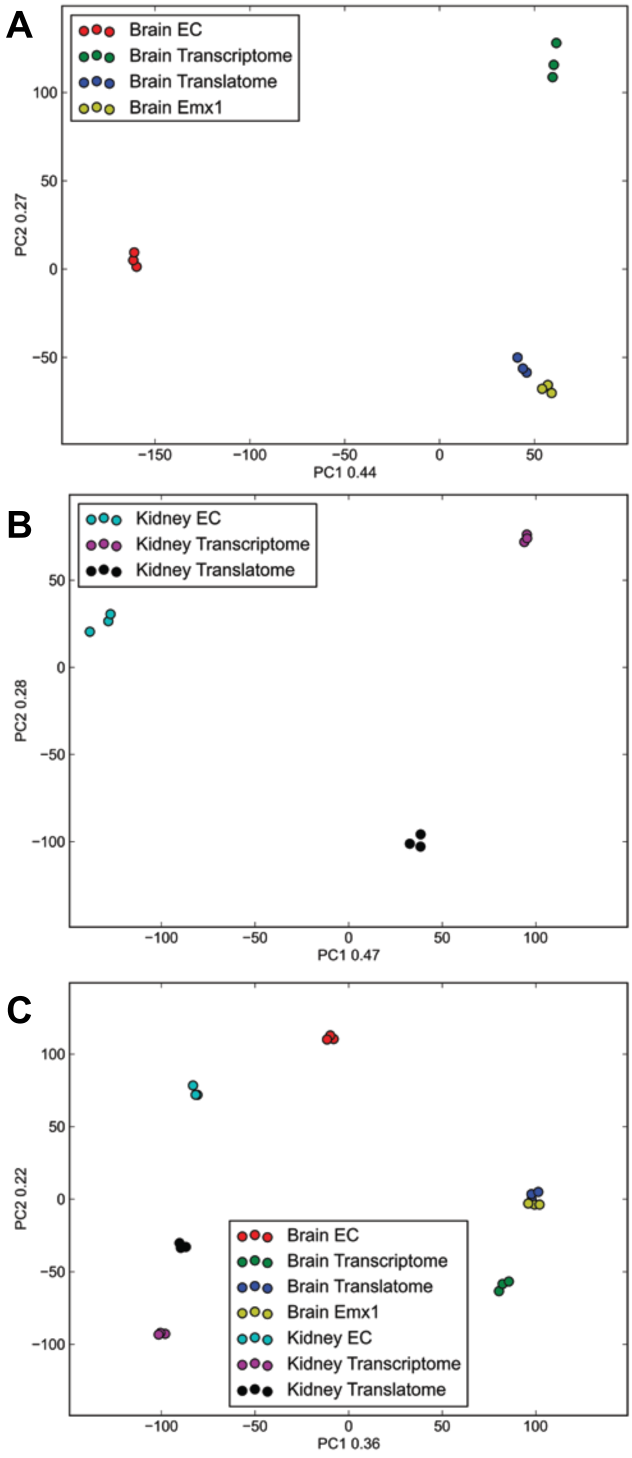

(**A**) Principal component analysis (PCA) analysis for all brain samples. (**B**) PCA for all kidney samples. (**C**) PCA for all brain and kidney samples. PC1 – first principal component. PC2 – second principal component. Note the clustering of Emx1 and Brain Translatome triplicate samples in A and C.

Supplementary Figure S7: Gene body coverage profiles

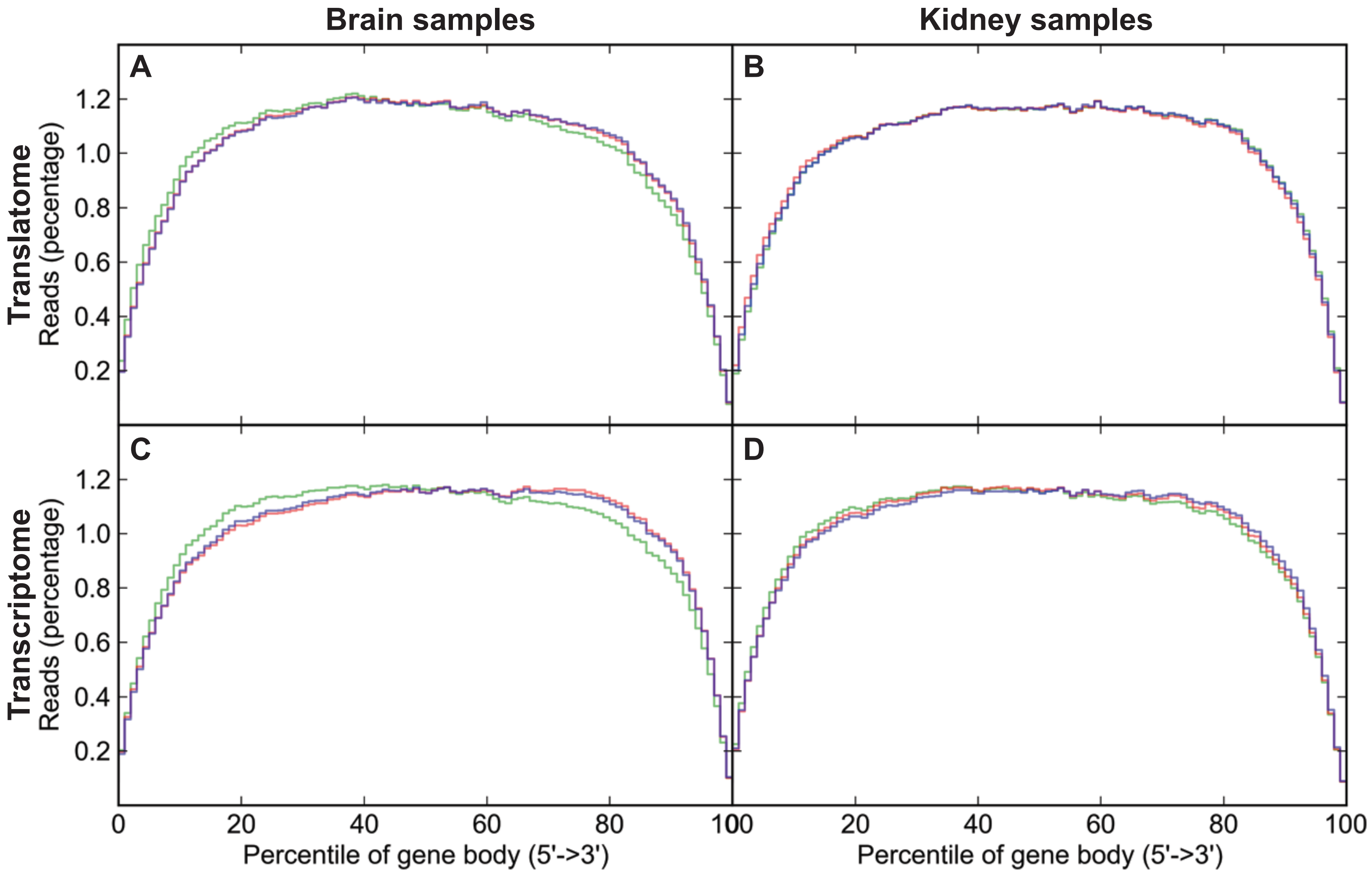

Similar gene body coverage profiles were obtained for translated (TRAP; **A**, **B**) and transcribed RNA (**C**, **D**) from *Sox2Cre+/-;R26-mCherry-Rpl10a+/-* E14.5 brains (**A**, **C**) and kidneys (**B**, **D**). The biological replicates are shown in red, blue, and green, respectively.

Supplementary Figure S8: Transcript length is a weak predictor of translational activity.

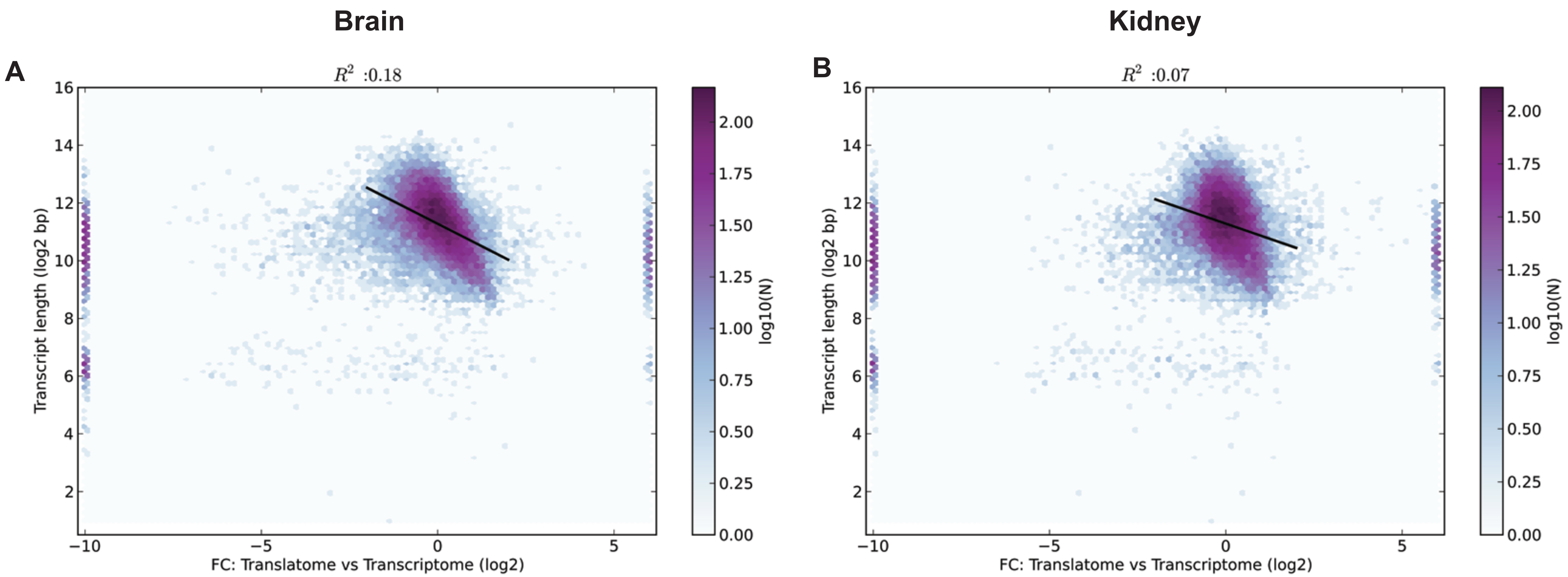

Hexagon binning of the fold change (FC) between translated (immunoprecipitated) and transcribed RNA from *Sox2Cre+/-;R26-mCherry-Rpl10a+/-* E14.5 forebrains (**A**) or kidneys (**B**) versus the length of the transcripts indicates that shorter transcripts are more highly translated than longer transcripts. Linear regression (-2 < FC < 2, transcript length > 7 (log2) bp) shows that transcript length is a weak predictor of translational activity.

Supplementary Figure S9: Differential expression of RefSeq non-coding RNAs between the translatome and transcriptome in the embryonic brain and kidney.

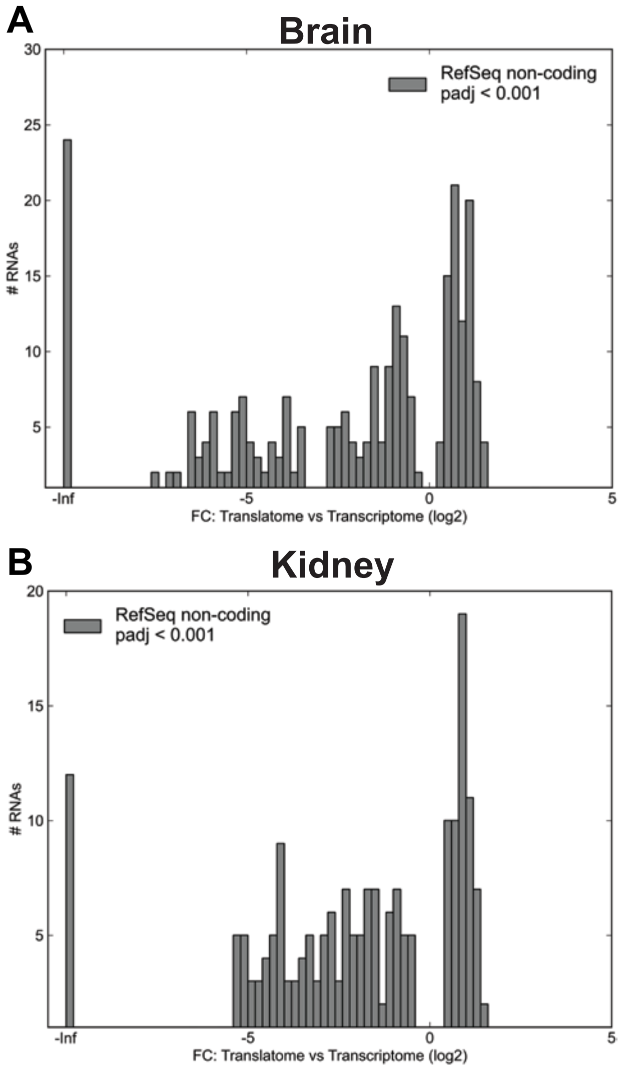

(**A**, **B**) Histograms showing the number of RefSeq non-coding RNAs that are either enriched (postitive fold change) or reduced (negative fold change) in the translatome versus the transcriptome of the E14.5 *Sox2Cre+/-;R26-mCherry-Rpl10a+/-* forebrain (**A**) or kidney (**B**). 85 RefSeq non-coding transcripts appear to be translated (FC > 0) in the brain (60 in kidney), indicating that they may be coding mRNAs.

Supplementary Figure S10: Evaluation of TRAP-seq by molecular profiling of kidney endothelial cells.

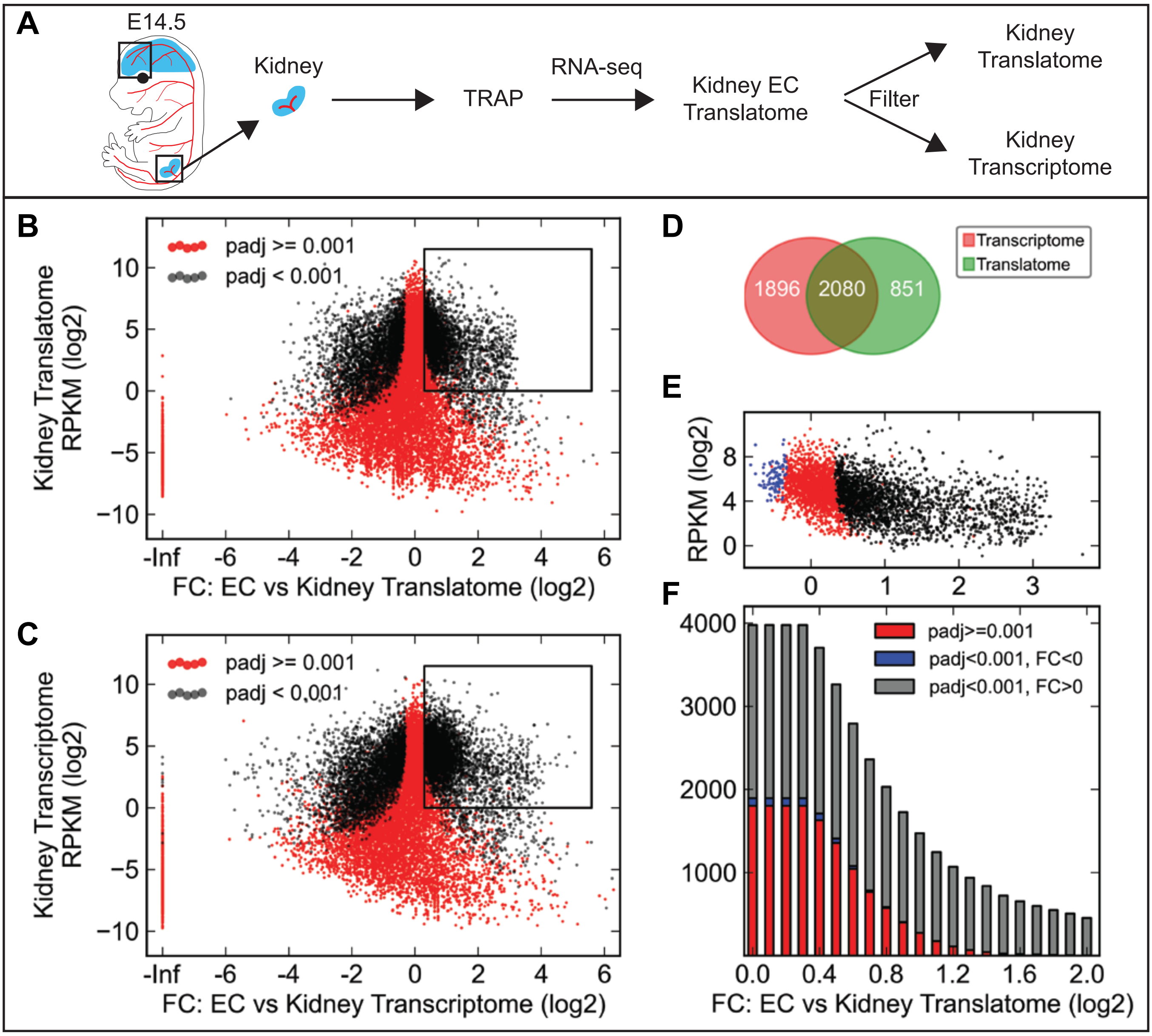

(**A**) Schematic of the experimental procedure to profile kidney endothelial cells. Two methods for identifying cell type specific transcripts are compared; using either translated or transcribed RNA from the kidney. Red color shows tissues expressing the mCherry-Rpl10a fusion protein. (**B**, **C**) Scatter plots of the fold change (FC) between immunoprecipitated endothelial cell (EC) RNA from E14.5 *Cdh5CreERT2+/-;R26-mCherry-Rpl10a+/-* kidneys and translated (**B**) or transcribed (**C**) RNA from entire *E14.5 Sox2Cre+/-;R26-mCherry-Rpl10a+/-* kidneys versus the average RPKM value for the transcript in the translatome (**B**) or transcriptome (**C**). Many transcripts are strongly enriched (positive fold change) in kidney endothelial cells. (**D**) Venn diagram showing the number of RNAs identified as enriched in kidney endothelial cells compared with the kidney translatome (box in **B**) or transcriptome (box in **C**) RNA (only RNAs above 1 RPKM). (**E**) All RNAs identified as kidney endothelial cell enriched in the comparison with the kidney transcriptome (**C**) were plotted using the fold change value obtained in the comparison with translatome RNA (**B**). Several RNAs were predicted by the transcriptome comparison to be enriched in endothelial cells, while in fact not significantly regulated (red, 1802 genes) or even reduced (blue, 94 genes). (**F**) A gradually increasing FC threshold was applied to the set of RNAs predicted to be enriched in kidney endothelial cells by the transcriptome comparison, and the number of RNAs that were (gray) or were not (red and blue) enriched also in the translatome comparison were quantified (see also Supplementary Figure S11).

Supplementary Figure S11: Transcriptome filtration is suitable for rare, but not for abundant, cell populations.

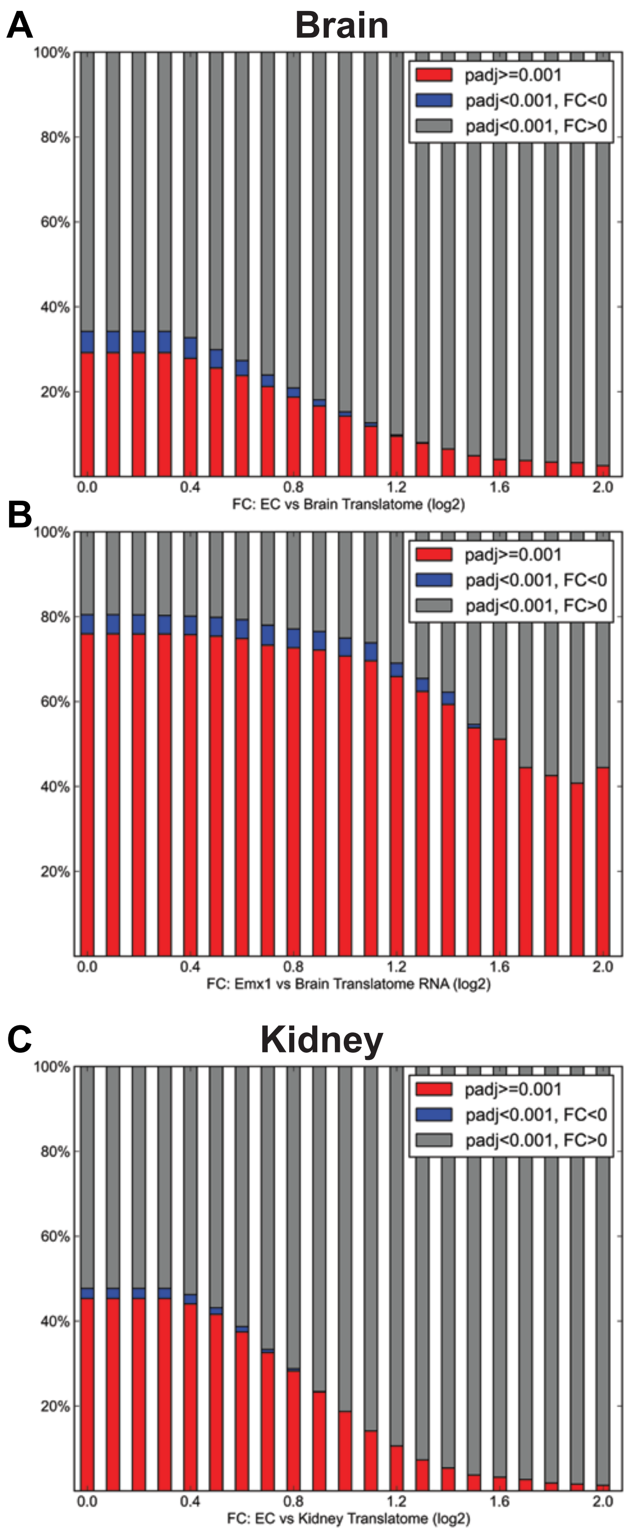

The data shown in the bar charts in Figures 3F, 4F, and Supplementary Figure 10F is here shown as the percentage (rather than absolute number) of RNAs predicted by the transcriptome comparisons to be enriched in (**A**) brain endothelial cells, (**B**) Emx1-positive dorsal telencephalon cells, and (**C**) kidney endothelial cells, that were (gray) or were not enriched (red and blue) also in the translatome comparison. Note that for the abundant Emx1 cell lineage, a very high percentage of transcripts were only predicted as cell type specific by the transcriptome comparison.

Supplementary Figure S12: Gene expression pattern of the top 25 (ranking 7-25) highest expressed transcripts identified as enriched in the Emx1-lineage versus the brain translatome.

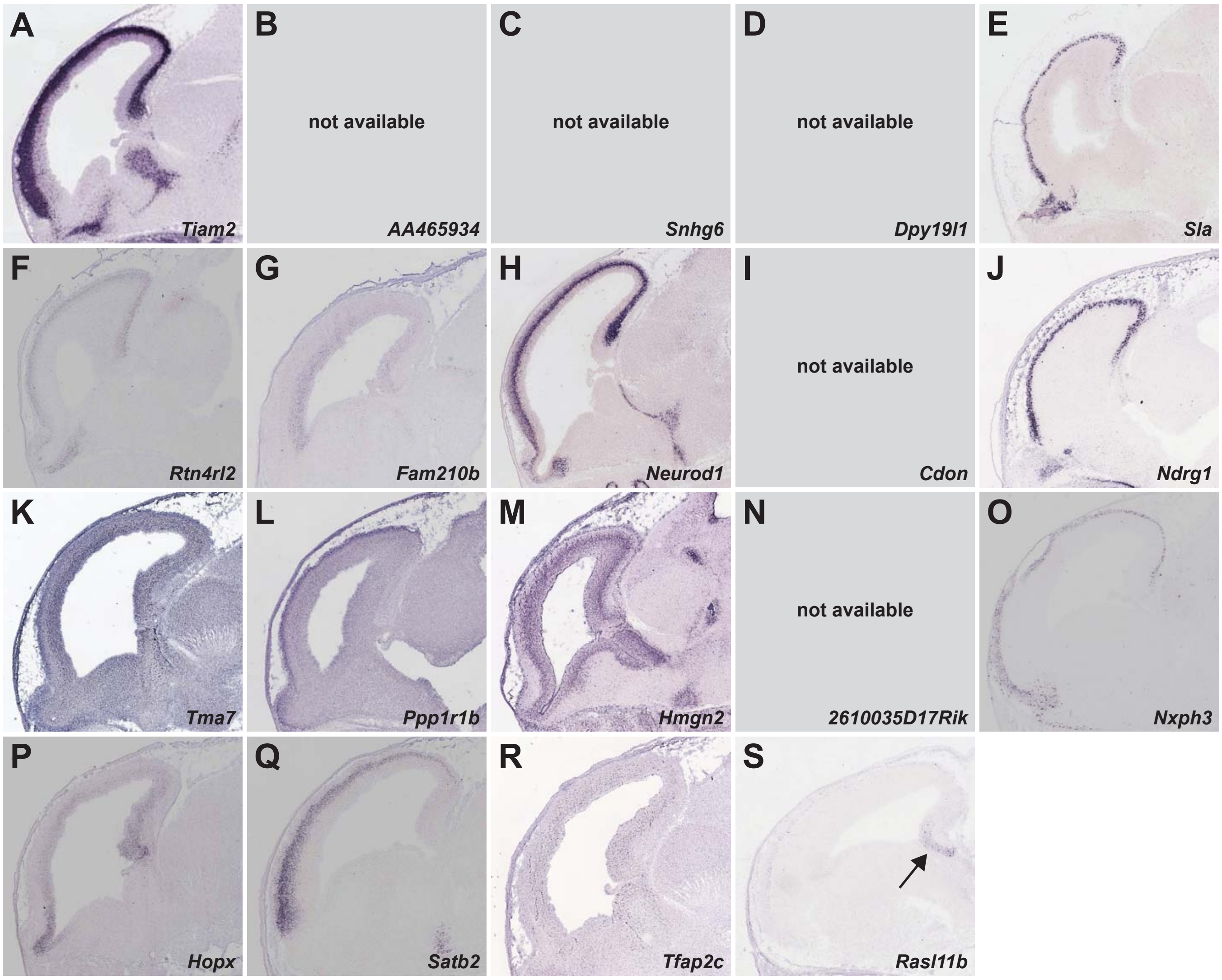

E14.5 gene expression data from Eurexpress (used with permission) is shown for the top 25 (ranking 7-25; for 1-6 see Figure 5) highest expressed genes identified as enriched in the Emx1-lineage versus the brain translatome (**A**-**S**) with a fold change > 2 (adjusted p-value < 0.001). Note that 7 transcripts are clearly enriched in the dorsal telencephalon (**E**, **H**, **J**, **O**-**Q, S**). 7 transcripts are not enriched in the dorsal telencephalon or are inconclusive (**A**, **F**, **G**, **K**-**M**, **R**). For 5 transcripts, there is no gene expression data in Eurexpress available (**B**-**D**, **I**, **N**).

Supplementary Figure S13: Gene expression pattern of the top 25 (ranking 7-25) highest expressed transcripts identified as enriched in the Emx1-lineage versus the brain transcriptome.

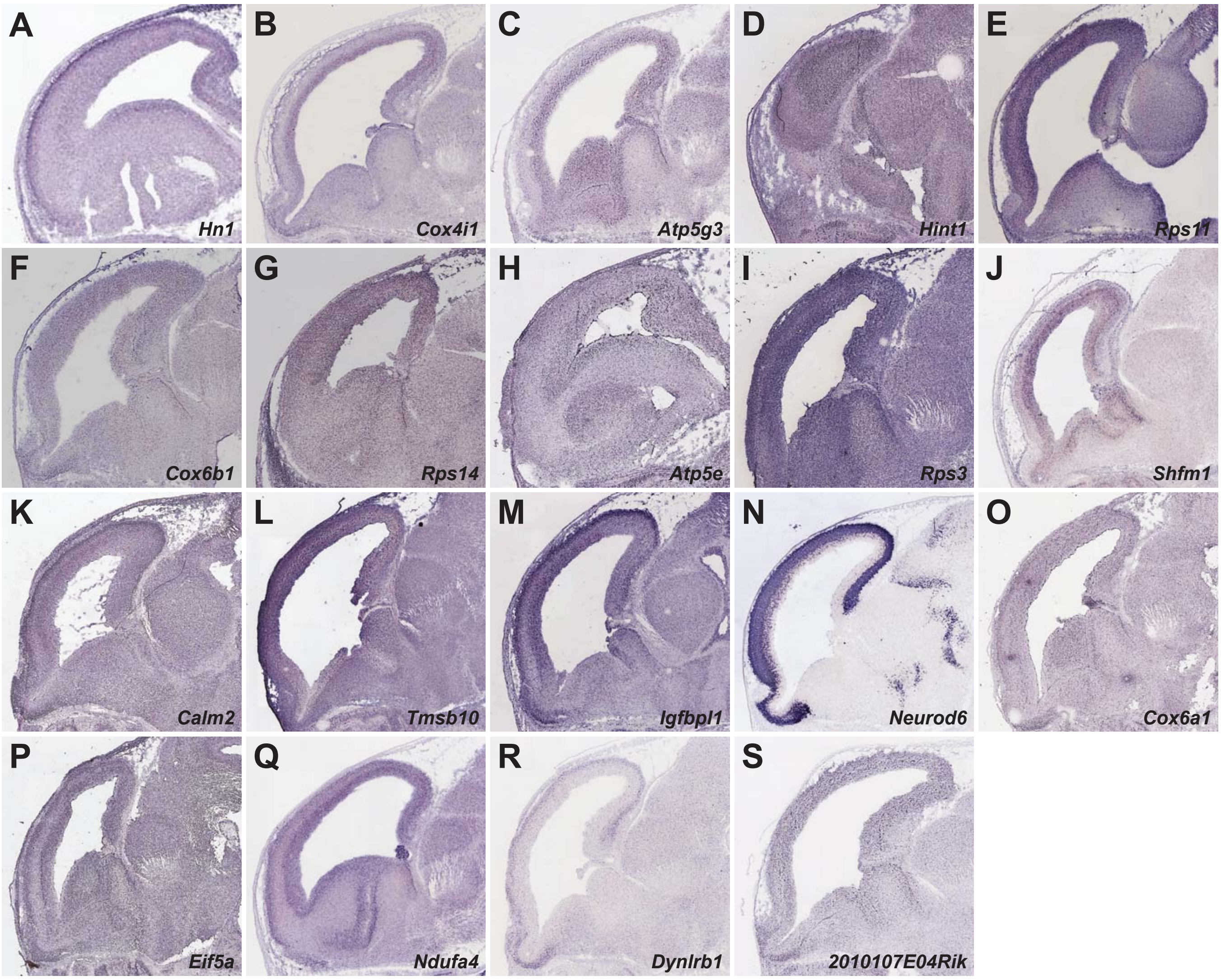

E14.5 gene expression data from Eurexpress (used with permission) is shown for the top 25 (ranking 7-25; for 1-6 see Figure 5) highest expressed genes identified as enriched in the Emx1-lineage versus the brain transcriptome (**A**-**S**) with a fold change > 2 (adjusted p-value < 0.001). Note that only 1 transcripts is clearly enriched in the dorsal telencephalon (**N**). All other transcripts are not enriched in the dorsal telencephalon or are inconclusive (**A**-**M**, **O**-**S**).

Supplementary Figure S14: Gene ontology (GO) analysis of RNAs determined to be expressed in brain and kidney endothelial cells.

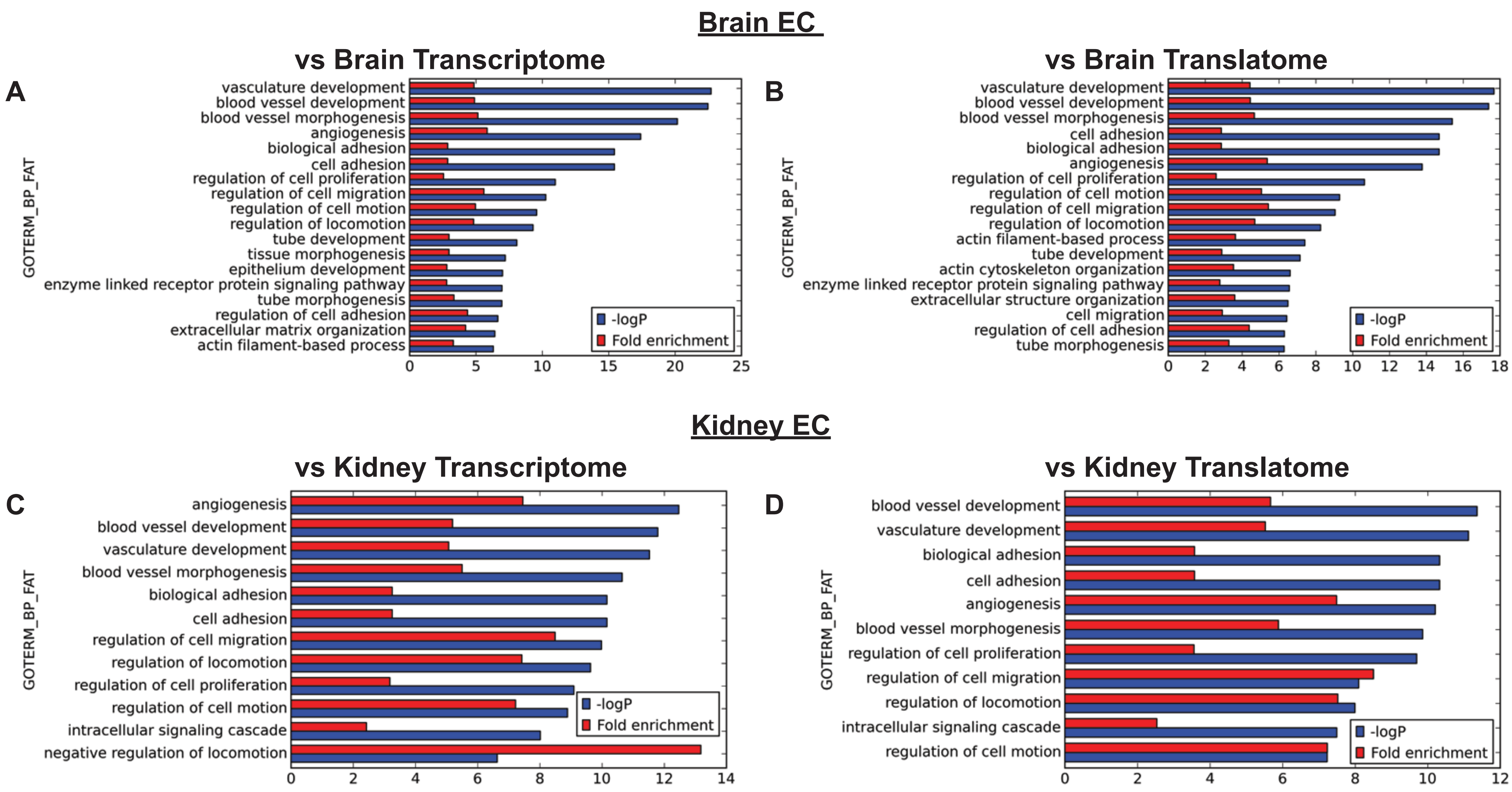

(**A**, **B**) Summary of the functional categories of transcripts identified to be enriched in brain endothelial cells versus the brain transcriptome (**A**) or translatome (**B**) RNA samples from E14.5 forebrains. (**C**, **D**) Summary of the functional categories of transcripts identified to be enriched in kidney endothelial cells versus the kidney transcriptome (**C**) or translatome (**D**) RNA samples from E14.5 kidneys. Analyses were performed using DAVID. All GO groups demonstrated enhanced statistical representation (FDR score < 0.001). Bars represent the –log10 EASE Score, a modified Fisher Exact P-Value, (blue) and the fold enrichment (red). For a complete breakdown of significantly enriched gene ontology groups, see Supplementary Tables S4-7.

Supplementary Figure S15: Validation of the applicability of TRAP-seq to study organ-specific kidney endothelial differentiation.

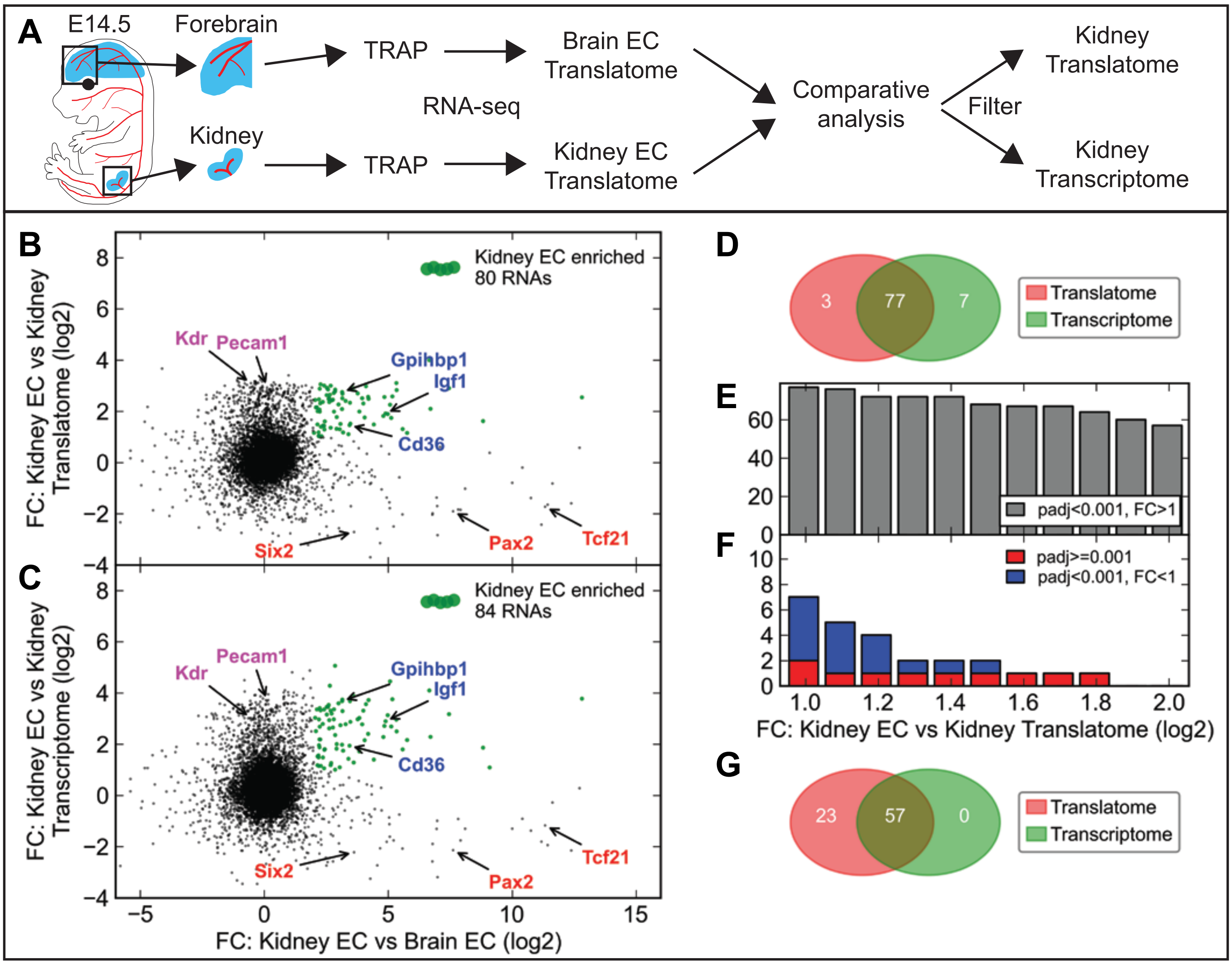

(**A**) Schematic of the experimental procedure to compare the molecular profile of kidney and brain endothelial cells. Red color shows tissues expressing the mCherry-Rpl10a fusion protein. (**B**, **C**) Scatter plots of the fold change (FC) between immunoprecipitated endothelial cell (EC) RNA from E14.5 *Cdh5CreERT2+/-;R26-mCherry-Rpl10a+/-* kidneys and forebrains versus the FC between kidney EC RNA and translated (**B**) or transcribed (**C**) RNA from E14.5 *Sox2Cre+/-;R26-mCherry-Rpl10a+/-* kidneys. RNAs that are at least twofold enriched versus the kidney translatome (**B**) or transcriptome (**C**), and fourfold enriched in kidney versus brain EC are indicated with green dots (n = 80 in **A**, n = 84 in **B**). General EC markers (magenta), general kidney markers (red), and three kidney enriched EC markers (blue) are indicated. (**D**) Venn diagram comparing the RNAs identified as kidney EC markers from the analyses in (**B** and **C**). 7 transcripts were predicted to be kidney EC enriched by the transcriptome, but not the translatome, analysis. (**E**, **F**) A gradually increasing FC threshold (kidney EC vs kidney transcriptome) was applied to the set of RNAs predicted to be kidney EC enriched by the transcriptome comparison, and the number of RNAs that were (gray in **E**) or were not (red and blue in **F**) enriched also in the translatome comparison were quantified. (**G**) A Venn diagram showing that with a FC threshold > 4, the number of transcripts identified as kidney enriched after transcriptome filtering have dropped from 84 to 57, all of which were also identified by the translatome comparison.
